# Cross‐Species Insights into Trophoblast Invasion During Placentation Governed by Immune‐Featured Trophoblast Cells

**DOI:** 10.1002/advs.202407221

**Published:** 2024-09-05

**Authors:** Xupeng Zang, Dan Zhang, Wenjing Wang, Yue Ding, Yongzhong Wang, Shengchen Gu, Yijun Shang, Jianyu Gan, Lei Jiang, Fanming Meng, Junsong Shi, Zheng Xu, Sixiu Huang, Zicong Li, Zhenfang Wu, Ting Gu, Gengyuan Cai, Linjun Hong

**Affiliations:** ^1^ State Key Laboratory of Swine and Poultry Breeding Industry, National Engineering Research Center for Breeding Swine Industry Guangdong Provincial Key Laboratory of Agro‐Animal Genomics and Molecular Breeding College of Animal Science South China Agricultural University Guangzhou 510642 P. R. China; ^2^ Reproductive Medicine Center Guangdong Provincial Key Laboratory of Reproductive Medicine The First Affiliated Hospital of Sun Yat‐sen University Guangzhou 510080 P. R. China; ^3^ Guangdong Key Laboratory of Animal Breeding and Nutrition Institute of Animal Science Guangdong Academy of Agricultural Sciences Guangzhou 510640 P. R. China; ^4^ Yunfu Subcenter of Guangdong Laboratory for Lingnan Modern Agriculture Yunfu 527300 P. R. China; ^5^ Key Laboratory of South China Modern Biological Seed Industry Ministry of Agriculture and Rural Affairs Guangzhou 510520 P. R. China

**Keywords:** iTrophoblast, placentation, preeclampsia, trophoblast invasion

## Abstract

Proper development of the placenta, the transient support organ forms after embryo implantation, is essential for a successful pregnancy. However, the regulation of trophoblast invasion, which is most important during placentation, remains largely unknown. Here, rats, mice, and pigs are used as biomedical models, used scRNA‐seq to comparatively elucidate the regulatory mechanism of placental trophoblast invasion, and verified it using a human preeclampsia disease model combined with scStereo‐seq. A dual‐featured type of immune‐featured trophoblast (iTrophoblast) is unexpectedly discovered. Interestingly, iTrophoblast only exists in invasive placentas and regulates trophoblast invasion during placentation. In a normally developing placenta, iTrophoblast gradually transforms from an immature state into a functional mature state as it develops. Whereas in the developmentally abnormal preeclamptic placenta, disordered iTrophoblast transformation leads to the accumulation of immature iTrophoblasts, thereby disrupting trophoblast invasion and ultimately leading to the progression of preeclampsia.

## Graphical abstract

Through cross‐species comparison, this study reveals the presence of a dual‐featured type of immune‐featured trophoblast (iTrophoblast) in invasive placentas, which regulates trophoblast invasion during placentation. Immature iTrophoblasts gradually transform into functional maturity with development, and their transformation disorder leads to the accumulation of immature iTrophoblasts, thereby disrupting trophoblast invasion and causing disease progression.

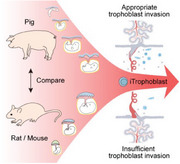

## 1 Introduction

The placenta serves as a transient support organ linking the mother and fetus, which is crucial to the success of pregnancy. Functionally, the placenta mediates the transport of nutrients, gases, and waste between mother and fetus, the production and uptake of hormones, and the regulation of maternal immune system to maintain normal fetal growth.^[^ ^1^, ^2^ ^]^ Similar to other organs, specialized trophoblast cells arising from multilineage cell differentiation pathways perform these functions cooperated with uterine cells.^[^ ^3^ ^]^ Most importantly, invasive trophoblast cells invade the uterine parenchyma and replace part of the vascular endothelium to remodel the uterine vasculature during placentation.^[^ ^4^, ^5^, ^6^ ^]^ Insufficient trophoblast invasion and defects in placental development frequently lead to severe obstetric syndromes, including intrauterine growth restriction and preeclampsia (PE).^[^ ^7^ ^]^ However, due to the structural complexity and developmental dynamics of the placenta, fully understanding the events that occur during placental development and associated pregnancy complications remains a huge challenge.

It is ethically and technically infeasible to study placentation after human embryo implantation, and most placenta samples are obtained during delivery in the third trimester.^[^ ^8^ ^]^ Because the depth and extent of intrauterine trophoblast invasion show significant differences between species,^[^ ^9^ ^]^ appropriate animal models are crucial to study placentation and related pathological triggers. Mice are the most common human biomedical models and are widely used in the study of various developmental biological events and diseases. However, the human placenta with the features of deep intrauterine trophoblast invasion is not possessed by mice.^[^ ^10^ ^]^ It is worth noting that rats have such deep trophoblast invasion features during placentation compared with mice, and are considered to be more suitable as a human reproductive model.^[^ ^11^ ^]^ Additionally, pigs are considered excellent large animal models of human health and disease due to their anatomical and physiological similarities to humans.^[^ ^12^ ^]^ However, pig placenta is indeed a non‐invasive epitheliochorial placenta, which is very different from human placenta.^[^ ^12^ ^13^ ^]^ This leaves open whether unique characteristics between species can be exploited to gain novel insights into placentation and trophoblast invasion.

The interaction between cells in uterus is indispensable for the function of the placenta. Thus, this study used single‐cell RNA sequencing (scRNA‐seq) technology to resolve important developmental events during rat and pig placentation and conduct complementary comparisons across species. We were able to precisely classify and define distinct subclusters of placental cells that exhibit dynamic changes during pregnancy. Particularly, we discovered a dual‐feature type of trophoblast with immune features in rats, named it immune‐featured trophoblast (iTrophoblast), and showed that it may play important regulatory roles in trophoblast invasion. Subsequently, using the human preeclampsia disease model, we combined with the single‐cell spatial enhanced resolution omics‐sequencing technology (scStereo‐seq) to verify the regulatory mechanism of trophoblast invasion obtained across species and highlighted the vital function of iTrophoblast during the trophoblast invasion process. Abnormal transformation of iTrophoblast could impair trophoblast invasion, thereby contributing to the progression of preeclampsia. These data provide a valuable resource for understanding in vivo placentation, regulation of trophoblast invasion, and the pathology and future treatment of preeclampsia.

## 2 Results

### 2.1 Decoding Single‐cell Transcriptome Profiles During Rat Placentation Using ScRNA‐seq

**Figure 1:**
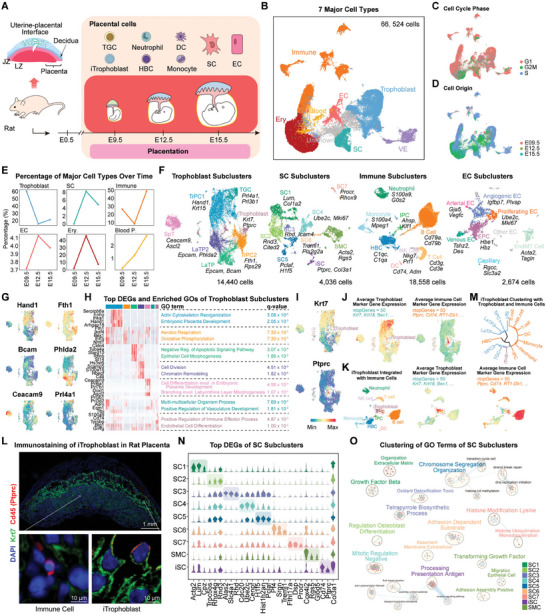
Decoding single‐cell transcriptome profiles during rat placentation using scRNA‐seq. A) Schematic diagram of the experimental design. The study included three critical stages of placental development in rats. The labyrinth zone (LZ) and junction zone (JZ) of rat placenta were collected. B) Uniform manifold approximation and projection (UMAP) visualization showing seven major cell types. C) UMAP visualization showing the cell cycle phase of rat placental cells. D) UMAP visualization showing the origin of placental cells at different stages. E) Line charts showing the proportion of major cell types in three stages. F) UMAP visualization showing subclusters of rat placental cell types. G) UMAP visualization showing marker gene expression in different placental trophoblast subclusters. H) Heatmap showing the top differentially expressed genes (DEGs) of trophoblast subclusters, and the GO biological process terms enriched by corresponding subcluster DEGs. I) UMAP visualization showing typical maker gene expression of trophoblast (*Krt7*) and immune (*Ptprc*) cell in clustered trophoblast subclusters. J,K) UMAP visualization showing the results of iTrophoblast integration with trophoblast and immune cell subclusters, and the corresponding average expression levels of trophoblast and immune cell marker genes. L) Immunostainings of Krt7 and Cd45 (Ptprc) protein in E15.5 rat placenta. M) Hierarchical clustering of iTrophoblast, trophoblast and immune cell subclusters. N) Violin plot showing the expression of top DEGs in SC subclusters. O) GO term clustering results of SC subclusters. E0.5‐E15.5, embryonic day 0.5 until 15.5; SC, stromal cell; EC, endothelial cell; Blood P., blood progenitor cells; Ery., erythrocyte; VE, visceral endoderm; TrPC, trophoblast progenitor cell; LaTP, labyrinth trophoblast progenitor cell; SpT, spongiotrophoblast; TGC, trophoblast giant cell; iTrophoblast, immune‐featured trophoblast cell; iSC, immune‐featured stromal cell; SMC, smooth muscle cell; IPC, immune progenitor cell; HBC, hofbauer cell; DC, dendritic cell; NK Cell, natural killer cell; EPC, endothelial progenitor cell; EndMT Cell, endothelial to mesenchymal transition cell.

Placentation is central to a successful pregnancy.^[^ ^1^ ^]^ However, the dynamics of rat placentation, which has a deep intrauterine trophoblast cell invasion more closely similar to humans,^[^ ^14^ ^]^ are not fully understood till today. In this study, we collected rat placenta samples from three critical stages of placentation (E9.5: the initial stage of placentation; E12.5: trophoblast cells remain in the placental junction zone; E15.5: deep intrauterine trophoblast cell invasion)^[^ ^11^, ^14^ ^]^ to perform 10X Genomics scRNA‐seq (**Figure** **1**A). After quality control, 66524 cells were retained for further analysis, and transcriptomes were then batch‐corrected across samples (Figure S1A, Supporting Information). First, we identified 7 major cell populations, including trophoblast, immune cells, stromal cells (SC), endothelial cells (EC), blood progenitor cells (Blood P.), erythrocyte (Ery.), and visceral endoderm (VE) (Figure 1B–D; Figure S1B and Table S1, Supporting Information). Additionally, we found that the cell composition of each stage during rat placentation is different (Figure 1E; Figure S1C, Supporting Information). The differences in the degree of trophoblast invasion in three stages reflect various biological events of trophoblast invasion to a certain extent.^[^ ^15^ ^]^

Next, we separately reclustered trophoblasts (14440 cells), SCs (4036 cells), immune cells (18558 cells), and ECs (2674 cells) to obtain detailed cell subclusters (Figure 1F; Table S1, Supporting Information). We first characterized the 7 trophoblast subclusters. The proportion of all identified trophoblast subclusters in rat placenta showed a dynamic change from E9.5 to 15.5 (Figure S1D, Supporting Information). Type 1 and 2 labyrinth trophoblast progenitor cells (LaTP and LaTP2) were enriched for epithelial cell morphogenesis and cell division (Figure 1F–H), suggesting the differentiation of potential syncytiotrophoblasts, which were not captured by 10X Genomics scRNA‐seq due to excessive cell diameter.^[^ ^16^, ^17^ ^]^ The spongiotrophoblast (SpT) was involved in placental labyrinth layer branching, whereas trophoblast giant cell (TGC) was associated with multicellular biological processes and the regulation of vascular development during placentation.^[^ ^18^, ^19^ ^]^ Notably, among the reclustered trophoblast cell population, we unexpectedly found a cell cluster expressing both typical immune cell (*Ptprc*) and trophoblast (*Krt7*) marker genes (Figure 1I–L). By using Scrublet,^[^ ^20^ ^]^ we ruled out the possibility that these cells were doublets (Figure S1E, Supporting Information). Transcriptome‐based hierarchical clustering suggested these cells were more related to trophoblast cells than immune cells (Figure 1M). Therefore, we named them as iTrophoblast, and their proportion in rat placenta is ≈1.40%.

For reclustered SCs, we defined 9 subclusters, including 7 SC subclusters, a population of SCs with immune‐related features (iSC), and smooth muscle cells (SMC) (Figure 1F). Each of the 7 SC populations expressed different specific genes (Figure 1N). Based on Gene Ontology (GO) enrichment analysis, SC1 subcluster (expressing *Lum*, and *Col1a2*) was involved in the “organization extracellular matrix” and was considered as stromal fibroblasts (Figure 1O).^[^ ^21^ ^]^ The SC3 subcluster, which was involved in “tetrapyrrole biosynthetic process”, represented the possible angiogenic SCs. The SC4 and SC5 subclusters were identified as proliferating SCs due to “mitosis regulation negative” and “chromosome segregation organization” being enriched. Interestingly, similar to recent studies, we also identified iSCs in the rat placenta (Figure 1F; Figure S1F–M, Supporting Information).^[^ ^21^ ^]^ Transcriptome‐based clustering and functional analysis showed that iSCs were likewise related to SCs but activated certain immune activities. The only difference is that we identified from the reclustered stromal cells, whereas previous studies of mouse implantation sites identified from reclustered immune cells.^[^ ^21^ ^]^

We also identified 8 immune cell subclusters, including neutrophils, monocytes, hofbauer cells (HBC), natural killer cells (NK), T cells, B cells, dendritic cells (DC), and immune progenitor cells (IPC) (Figure 1F; Figure S1N, Supporting Information). For EC, we detected 7 subclusters. These ECs in the placenta were involved in vasculogenesis and cell migration, whereas angiogenic ECs and proliferating ECs were associated with sprouting angiogenesis (Figure 1F; Figure S1O, Supporting Information).^[^ ^22^ ^]^ The discovery of endothelial to mesenchymal transition cells (EndMT) suggested that endothelial‐mesenchymal transition may exist during rat placentation.^[^ ^23^ ^]^ EndMT plays an important role in regulating cell invasion and metastasis.^[^ ^24^ ^]^

Thus, by using 10X Genomics scRNA‐seq technology, we were able to characterize the dynamics of different placental cell types during rat placentation, which allowed us to study key events in developmental regulation at the single‐cell transcriptome level.

### 2.2 Constructing of the Single‐Cell Transcriptome Atlas During Early Placentation in Pigs

**Figure 2:**
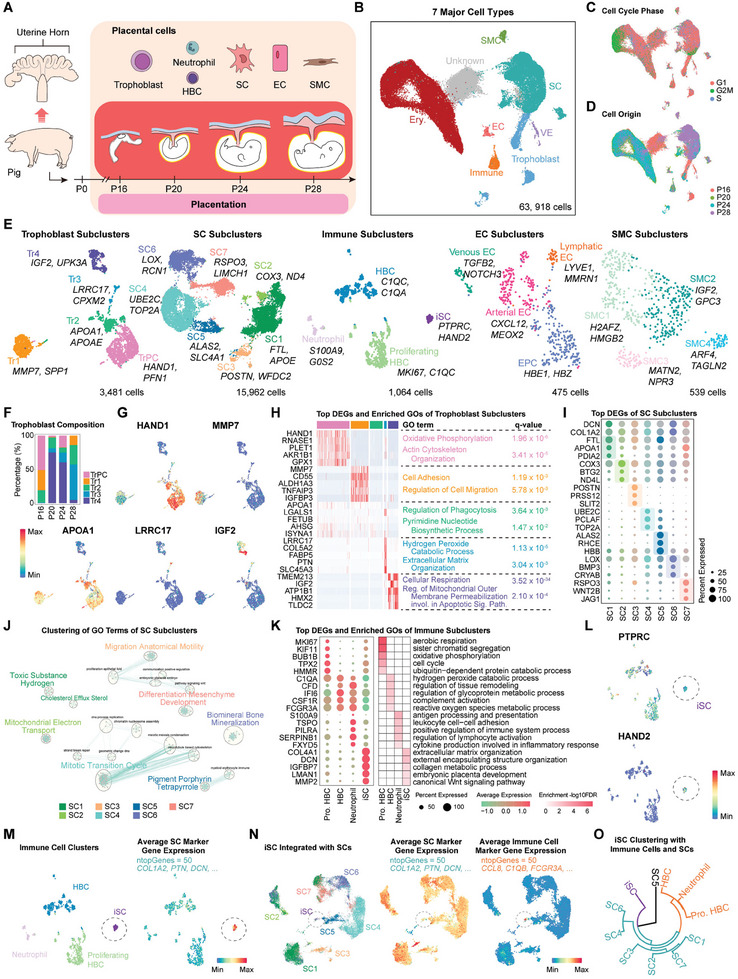
Constructing of the single‐cell transcriptome atlas during early placentation in pigs. A) Schematic diagram of experimental design. The study included four different stages of early placentation in pigs. B) UMAP visualization showing seven major cell types of pig placenta. C) UMAP visualization showing the cell cycle phase. D) UMAP visualization showing the origin of placental cells at different stages. E) UMAP visualization showing subclusters of pig placental cell types. F) Stacked bar plot showing the proportion of each trophoblast subcluster in four stages. G) UMAP visualization showing marker gene expression in different placental trophoblast subclusters. H) Heatmap showing the top DEGs of trophoblast subclusters and the enriched GO terms by corresponding DEGs. I) Dot plot showing the expression of top DEGs in SC subclusters. J) GO term clustering results of SC subclusters. K) Dot plot showing the top DEGs in immune cell subclusters, and the heatmap showing the enriched GO terms. L) UMAP visualization showing typical maker gene expression of immune (*PTPRC*) and stromal (*HAND2*) cell in clustered immune cell subclusters. M,N) UMAP visualization showing the results of iSC integration with immune cell and SC subclusters, and the corresponding average expression levels of immune cell and SC marker genes. O) Hierarchical clustering of iSC, SC and immune cell subclusters. P0‐P28, day 0 to 28 of pregnancy.

Unlike the deep trophoblast‐invasive hemochorial placenta of rats, pigs use a non‐invasive epitheliochorial placenta.^[^ ^13^ ^]^ This led us to speculate whether the regulation of trophoblast invasion could be studied by comparing differences in rat and pig placentation. However, a comprehensive understanding of placentation in pigs has also not been achieved till today. Considering the substantial differences in placental types between species, and pig placenta will gradually form a folded structure after day 30 of pregnancy, this study only collected pig placenta samples during early placentation (before day 30 of pregnancy) to perform 10X Genomics scRNA‐seq (**Figure** **2**A).^[^ ^25^ ^]^ After quality control, 63918 pig placenta cells were retained for subsequent analysis, and the transcriptomes were then batch‐corrected (Figure S2A, Supporting Information). We similarly identified 7 major cell populations, including trophoblast, immune cells, SCs, ECs, Ery., VE, and SMCs, but no Blood P. (Figure 2B–D; Figure S2B,C and Table S2, Supporting Information).

Next, we reclustered trophoblast cells (3481 cells), SCs (15962 cells), immune cells (1064 cells), ECs (475 cells), and SMCs (539 cells) to obtain detailed cell subclusters (Figure 2E; Table S2, Supporting Information), respectively. In early pig placenta, we identified a total of 5 different epitheliochorial trophoblast subclusters, each expressing different specific genes (Figure 2E–G). Type 1 epitheliochorial trophoblast subcluster (Tr1) was enriched in cell adhesion and migration, and mainly existed in P16, suggesting a population was involved in the implantation of pig embryos.^[^ ^26^ ^]^ The Tr2 subcluster was involved in the “regulation of phagocytosis” and was considered the areola unique to pig placenta, participating in the transport of placental nutrients.^[^ ^27^, ^28^ ^]^ The cell proportion of Tr3 subcluster gradually increases during placentation and was associated with “extracellular matrix organization”, which may be involved in the formation of epitheliochorial placental ridges.^[^ ^26^, ^29^ ^]^

For reclustered SCs, 7 SC subclusters were defined, and we found that some of them were similar to rat SC subclusters, while others were unique to pigs (Figures 1F and 2E,I). The SC4 subcluster in pig placenta involved in the “mitotic transition cycle” was identified as proliferating SCs similar to the SC4 and SC5 subclusters identified in rats, whereas the SC5 subcluster involved in “pigment porphyrin tetrapyrrole” similar to the rat SC3 subcluster represented the angiogenesis SCs (Figure 1O; Figure S2J, Supporting Information). Other defined SC subclusters, such as the SC2 subcluster, were involved in energy metabolism. SC3 and SC7 subclusters were related to “migration anatomical motility” and “differentiation mesenchyme development”, which may cooperate with the trophoblast Tr3 subcluster to form the epitheliochorial placental ridges, while the SC6 subcluster was considered as the biomineral‐regulatory SCs.^[^ ^21^ ^]^

Among immune cells, in addition to iSCs, we identified only 3 immune cell subclusters in pig placenta, including neutrophils, HBCs, and proliferative HBCs (Figure 1E,K). Notably, this population of iSCs derived from reclustered immune cell subclusters was from the same origin as the study in mouse implantation sites,^[^ ^21^ ^]^ and was different from the iSCs identified in rat placenta mentioned above (Figure 2L–N; Figure S2E, Supporting Information). Transcriptome‐based hierarchical clustering showed that iSCs remain related to SCs, which appears to be independent of the origin of iSCs identified (Figure 2O). However, functional analysis of differentially expressed genes (DEGs) between iSCs and SCs revealed that there are still some differences in the roles of iSCs in different species. For example, iSCs in rat placenta regulated vasculature development but not in pigs (Figures S1K,L and S2F,G, Supporting Information).

For EC, we detected 4 subclusters, including arterial ECs, venous ECs, lymphatic ECs, and endothelial progenitor cells (EPC) (Figure 2E; Figure S2H, Supporting Information). These ECs in pig placenta were involved in the development and maturation of placental blood vessels.^[^ ^30^ ^]^ Additionally, we identified 4 SMC subclusters. SMC1 subcluster expressed TOP2A and was enriched in the “cell cycle”, represented a population of proliferating SMC. The SMC2 subcluster was involved in processes of energy metabolism, whereas the SMC3 subcluster was in “collagen fibril organization” and “epithelial tube branching” (Figure 2E; Figure S2I, Supporting Information).

### 2.3 Cross‐species Comparative Analysis of Placental Cells During Placentation

**Figure 3:**
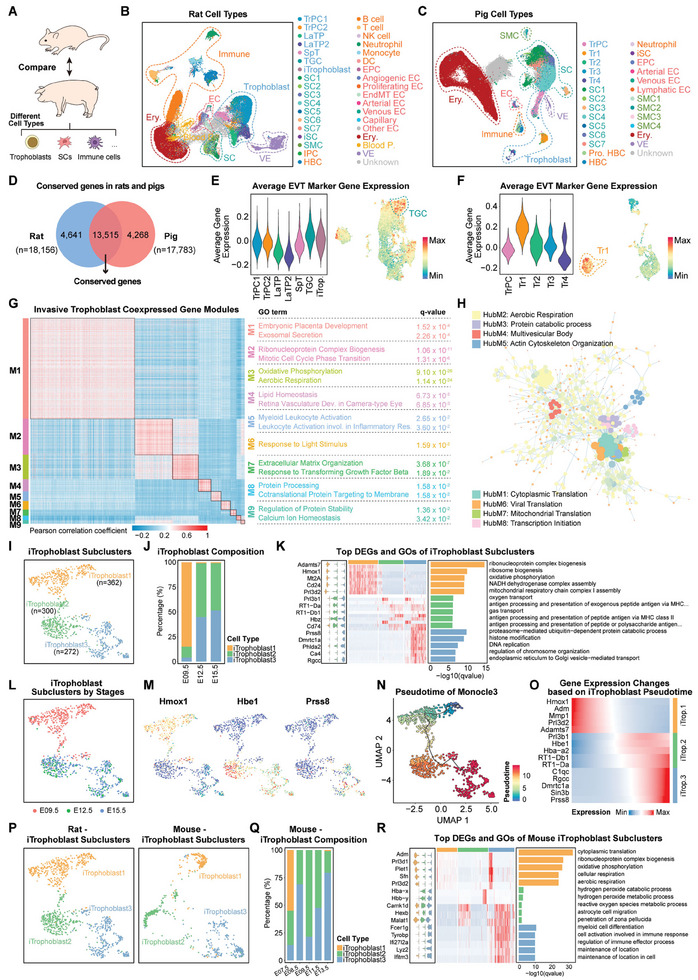
Cross‐species comparative analysis of placental cells during placentation. A) Schematic diagram of cross‐species comparison of rat and pig placental cells. B,C) UMAP visualization showing all subclusters of major cell types identified in rat and pig placenta. D) Conserved genes identified in rat and pig placental cells. E,F) Violin plot and UMAP visualization showing the average expression levels of top 50 extravillous trophoblast cell (EVT) marker genes in rat and pig different trophoblast subclusters. G) Heatmap showing correlation of coexpressed gene modules in invasive trophoblast generated by WGCNA in rat and pig placentas. GO terms related to the module are highlighted on the right. H) PPI network of M1 module genes. Highlighting the 8 hub modules identified. I) UMAP visualization showing the reclustering of iTrophoblast cells. J) Stacked bar plot showing the proportion of each iTrophoblast subcluster in three stages. K) Violin plots and heatmap showing the top DEGs of iTrophoblast subclusters and enriched GOs. L) UMAP visualization showing the origin of iTrophoblast cells at different stages. M) UMAP visualization showing marker gene expression in different iTrophoblast subclusters. N) Predicted trajectories of iTrophoblast colored with pseudotime. O) Heatmap showing the clustering of DEGs along iTrophoblast developmental trajectory. P) UMAP projection of mouse iTrophoblasts showing the three subclusters of iTrophoblasts. Q) Stacked bar plot showing the proportion of mouse iTrophoblast subcluster in five stages. R) Violin plots and heatmap showing the top DEGs of mouse iTrophoblast subclusters and enriched GOs.

To better understand regulatory differences during placentation, we performed a comprehensive comparative analysis in rats and pigs (**Figure** **3**A). Meanwhile, we downloaded and analyzed data on mouse placentation from public databases^[^ ^16^ ^]^ (Figure S3 and Tables S4 and S8, Supporting Information). Overall, in this study, we mainly identified 34 different types of rat placental cells and 26 types of pig placental cells, including cells common to both species, unique to rats, and unique to pigs (Figure 3B,C). Additionally, due to the obvious differences in genes expressed by different species cells, homologous genes play an indispensable role in cross‐species comparative analysis.^[^ ^31^ ^]^ For our 10X Genomics single‐cell transcriptome of placental cells, a total of 13515 homologous genes in rats and pigs were identified for subsequent analysis (Figure 3D).

Referring to previous methods,^[^ ^32^ ^]^ we calculated the invasiveness index of all trophoblast cells in rat and pig placenta using the identified marker genes of invasive trophoblast cells. As expected, invasive TGCs in rats had the highest trophoblast invasiveness index, which was consistent with the performance of TGCs in mouse placenta (Figure 3E; Figure S3N, Supporting Information).^[^ ^33^ ^]^ To our surprise, the Tr1 subcluster in pig trophoblast also acquired a high trophoblast invasiveness index (Figure 3F). Based on the above functional analysis of Tr1 subcluster (Figure 2H), we speculate that the invasion of this trophoblast population contributes to the close apposition of placental tissue and endometrial cells, thereby shortening the interhaemal distance.^[^ ^34^ ^]^ Accordingly, we hoped to gain insights into the regulatory mechanisms of trophoblast invasion by comparing the different expression patterns of the identified homologous genes in rat TGC and pig Tr1 along their respective developmental trajectories.

We analyzed 13515 homologous genes in TGCs and Tr1 and identified 9 distinct gene expression modules (M1‐M9) by weighted gene co‐expression network analysis (WGCNA) (Figure 3G; and Table S3, Supporting Information). Genes in these modules had different gene expression patterns in TGCs and Tr1 with placentation and performed distinct biological functions (Figure 3G; Figure S4A,B, Supporting Information). M1 contained the largest number of genes involved in placental development and was identified as a functional gene module for invasive trophoblast as the gene expression in TGCs and Tr1 gradually increases with placentation. Genes in M2 were involved in the “mitotic cell cycle phase transition” and expressed at higher levels in TGCs but at lower levels in Tr1, which was related to the transition from the mitotic to the endoreduplication cell cycle during TGC development.^[^ ^19^ ^]^ Genes in M4 and M5 were barely expressed in rat TGCs, but had higher levels in pig Tr1, suggesting that unique functional modules in pig invasive trophoblast were involved in angiogenesis and inflammatory responses.^[^ ^30^ ^]^ Next, we performed protein‐protein interaction (PPI) analysis on the genes in functional module M1 to identify hub module genes and constructed a regulatory network based on 8 hub modules (Figure 3H; and Table S3, Supporting Information). These 8 hub modules linked different genes to regulate different functions, thereby regulating the development of invasive trophoblast.

Most notably, we found that iTrophoblast were identified only in the invasive hemochorial placenta of rats and mice, but not in the non‐invasive epitheliochorial placenta of pigs (Figure 3B,C; Figure S3G, Supporting Information). Does this mean that iTrophoblast play a crucial role in invasive placenta formation? Therefore, we further reclustered the iTrophoblast population obtained in rats and identified 3 subclusters with distinct transcriptome features (Figure 3I–K; and Table S1, Supporting Information). Interestingly, we found that the cell composition of these 3 iTrophoblast subclusters at different stages showed stepwise changes with placental development (Figure 3J,L,M). To precisely understand the dynamic processes of iTrophoblast subclusters, we performed Monocle3 pseudotime analysis. As expected, this analysis revealed that iTrophoblast1 first converted into iTrophoblast2 and then transformed into iTrophoblast3 (Figure 3N,O). Unlike rats, where iTrophoblasts were identified only from reclustered trophoblasts, two iTrophoblast populations were identified in the mouse placenta, originating from reclustered trophoblasts and immune cells, respectively, with a proportion of ≈2.60% (Figure S3G,O and Table S4, Supporting Information).^[^ ^16^ ^]^ By comparing these two iTrophoblast populations, we found that iTrophoblast origin from reclustered immune cells were mostly derived from the early placenta (E7.5–E9.5) and were defined as immature iTrophoblast, whereas iTrophoblast origin from reclustered trophoblast cells were mostly derived from the placenta of later developmental stages (E11.5–E13.5), defined as mature iTrophoblast (Figure S3G, Supporting Information). Monocle pseudotime analysis revealed the transition of immature iTrophoblast to mature iTrophoblast (Figure S3P, Supporting Information). The identification of intermediate transition genes suggested that iTrophoblast formation involves cell‐cell fusion (Figure S3Q, Supporting Information).

To investigate whether the difference in the degree of intrauterine trophoblast invasion between rat and mouse placentas could be explained by the identified iTrophoblast, we projected the iTrophoblast identified in mouse placentas onto different subclusters of rat iTrophoblast. Three mouse iTrophoblast subclusters were also identified, with iTrophoblast1 being the initiating subcluster of developmental transformation, but more dispersed compared with rats (Figure 3P,Q). Comparison of the enriched GO biological processes in three trophoblast subclusters revealed that the iTrophoblast1 subcluster in rat and mouse placenta played similar roles. In rats, the transformed iTrophoblast2 subcluster had begun to perform some immune responses, while in mouse placenta, it was not until iTrophoblast3 that a similar immune response was exerted (Figure 3K,R), which may ultimately lead to the weaker trophoblast invasion ability of mouse placenta compared to rat.

Since recent studies have shown that iSCs play an important role in immune cell recruitment and angiogenesis, which is also critical for placental trophoblast invasion.^[^ ^21^, ^35^, ^36^ ^]^ Thus, we compared iSCs in rats and pigs. Unfortunately, through WGCNA, the co‐expressed gene modules of iSCs were not as distinct as those of invasive trophoblasts (Figure S4C,D, Supporting Information). Interestingly, we identified iSCs from reclustered SCs in mouse placenta, which is consistent with the origin of iSCs we identified in rat placenta, but different from pig placenta and mouse implantation sites,^[^ ^21^ ^]^ which were identified from reclustered immune cells. Based on the above understanding of iTrophoblast, we speculated whether the iSCs identified in pig placenta and mouse implantation sites were immature iSCs, while the iSCs in rat and mouse placentas were mature iSCs that played certain roles in trophoblast invasion. We integrated iSCs from mouse implantation sites and mouse placenta and performed monocle analysis. The results were consistent with iTrophoblast, revealing the transition of immature iSCs into mature iSCs, but the formation mechanism of iSCs may be different from iTrophoblast because no obvious biological process of cell‐cell fusion has been identified (Figure S3S,T, Supporting Information). In addition, due to the important functions of SCs in placental development, the SC1 subcluster in rats and the SC3 subcluster in pigs were enriched in biological processes such as extracellular matrix organization and migration anatomical motility (Figures 1O and 2J), respectively, which may play a pivotal role in helping trophoblast migration and invasion. We analyzed homologous genes in rat SC1 and pig SC3 and identified 10 distinct gene expression modules (M1‐M10) by WGCNA (Figure S4E, Supporting Information). The genes in these SC modules also had different gene expression patterns and performed biological functions such as cell migration and immune cell recruitment (Figure S4F–H, Supporting Information).

In summary, by comparing single‐cell transcriptomic data during rat and pig placentation, we identified some potential regulatory mechanisms of trophoblast invasion.

### 2.4 Functional Cellular Disorders Regulating Trophoblast Invasion of Placenta in Human Preeclampsia

**Figure 4:**
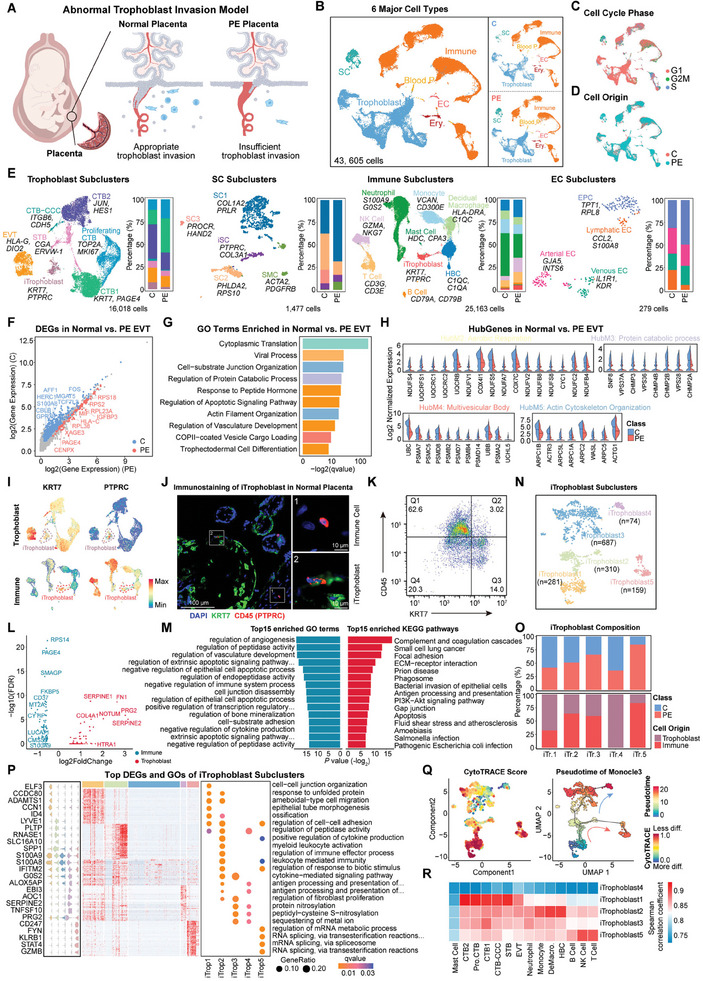
Functional cellular disorders regulating trophoblast invasion of placenta in human preeclampsia. A) Schematic diagram of human normal and preeclamptic placental sample collection. B) UMAP visualization showing the six major cell types in human normal (C) and preeclamptic (PE) placenta. C) UMAP visualization showing the cell cycle phase of human placental cells. D) UMAP visualization showing the origin of placental cells in both samples. E) UMAP visualization showing subclusters of human placental cell types, with a stacked bar plot showing the proportion corresponding to each subcluster. F) Scatter plot showing DEGs of PE placenta compared with normal placenta in EVT. G) GO enrichment terms of DEGs of PE placenta compared with normal placenta in EVT. H) Violin plots showing gene expression of hub modules 2–5 identified above in normal and PE placental EVT. I) UMAP visualization showing typical marker gene expression of trophoblast (*KRT7*) and immune (*PTPRC*) cells in clustered trophoblast and immune cell subclusters. J) Immunostainings of KRT7 and CD45 (PTPRC) protein in human normal placenta. K) Flow cytometry analysis of iTrophoblast expressing KRT7 and CD45 (PTPRC) in normal human placenta. L) Volcano plots showing DEGs between mature and immature iTrophoblast subclusters. M) GO enrichment and Kyoto Encyclopedia of Genes and Genomes (KEGG) pathway analysis of DEGs between mature and immature iTrophoblast subclusters, showing the top 15 enriched terms for each category. N) UMAP visualization showing the reclustered iTrophoblast subclusters. O) Stacked bar plot showing the proportion of each iTrophoblast subcluster in different placenta samples and subcluster origins. P) Heatmap showing the top DEGs of iTrophoblast subclusters and enriched GO terms. Q) Cell differentiation score calculated by CytoTRACE and predicted trajectories of iTrophoblast colored by pseudotime. R) Heatmap showing the correlation between iTrophoblast subclusters with different types of trophoblast and immune cells. C, normal placenta; PE, preeclamptic placenta; CTB, cytotrophoblast; CTB‐CCC, cytotrophoblast cell column‐cytotrophoblast; STB, syncytiotrophoblast; EVT, extravillous trophoblast.

Human preeclampsia provides an excellent disease model to validate our cross‐species identification of regulatory mechanisms for trophoblast invasion, characterized by inadequate extravillous trophoblast invasion and spiral artery remodeling (**Figure** **4**A).^[^ ^37^, ^38^ ^]^ Thus, we collected normal and preeclamptic placenta samples to apply scRNA‐seq while integrating data from previous public databases (Tables S7 and S8, Supporting Information).^[^ ^39^ ^]^ Finally, we obtained 43605 high‐quality human placenta cells and identified 6 major cell populations including trophoblast, immune cells, SCs, ECs, Ery., and Blood P., without VE in early placentation (Figure 4B–D; Figure S5A–C and Table S5, Supporting Information). Unsupervised reclustering analysis further demonstrated comparable placenta cell subclusters across species, also human normal and PE placentas (Figure 4E; Figure S5D–I and Table S5, Supporting Information). Similar to previous studies,^[^ ^40^, ^41^ ^]^ the PE placenta exhibited a lower proportion of extravillous trophoblast cell (EVT) compared with normal placenta (Figure 4E). Transcriptome comparison showed that EVT displayed significant differences between PE and normal placenta (Figure 4F). Interestingly, DEGs in the EVT played similar biological roles to the functional module M1 identified in above comparison of rat and pig invasive trophoblasts (Figures 3H and 4F,G). To our great surprise, the expression levels of all genes in the eight hub modules identified in M1 were reduced to varying degrees in PE placental EVT compared with normal placenta (Figure 4H; Figure S5J, Supporting Information). These findings suggest a functional disorder of EVT during preeclampsia.

What excites us most is that we also found iTrophoblast in the human placenta, with the proportion of iTrophoblast being ≈3.47% (Figure 4E,I,J; Figure S5K, Supporting Information), which is an invasive hemochorial placenta. Flow cytometry also confirmed the rare proportion of iTrophoblast in the human placenta (Figure 4K; Figure S5L, Supporting Information). And we identified two iTrophoblast populations in normal and PE placentas as in mouse placentation (Figure S3G,O, Supporting Information). Among them, iTrophoblast origin from reclustered trophoblast cells had an extremely high proportion in the normal placenta, corresponding to the defined mature iTrophoblast; while iTrophoblast from reclustered immune cells had a very high proportion in PE placenta, corresponding to immature iTrophoblast. Comparing the transcriptome differences between mature and immature iTrophoblast subclusters, these identified DEGs were significantly enriched in biological processes such as the regulation of angiogenesis and immune system, and cell‐substrate adhesion (Figure 4L,M). While biological processes such as cell adhesion and leukocyte migration were also enriched in the DEGs of mature iTrophoblast and EVT subclusters (Figure S5M,N, Supporting Information), suggesting that mature iTrophoblast could regulate trophoblast invasion.

Since trophoblast invasion occurs early in pregnancy, we downloaded and analyzed data on placental villi and decidual at the human first‐trimester maternal‐fetal interface from public databases^[^ ^42^, ^43^ ^]^ (Figures S6 and S7 and Tables S6 and S8, Supporting Information). Notably, we identified both immature and mature iTrophoblasts in the placental villi, but only mature iTrophoblasts in the decidua, suggesting that these mature iTrophoblasts had invaded deep into the placenta along with trophoblasts. Considering the putative key roles of iTrophoblast in invasive placentation, we propose that maybe excess immature iTrophoblast leads to insufficient trophoblast invasion during placentation in preeclampsia patients, thereby contributing to the development of preeclampsia.^[^ ^44^ ^]^

To further understand the iTrophoblast mechanism, we reclustered iTrophoblast cells obtained from the human placenta and ultimately identified five subclusters with distinct transcriptome profiles (Figure 4N–P; Figure S5O, Supporting Information). Interestingly, we found that iTrophoblast4 only exists in mature iTrophoblast, and its proportion in normal placenta is higher than in PE placenta; while iTrophoblast5 has a remarkably high proportion in immature iTrophoblast and PE placenta (Figure 4O). CytoTRACE‐based and Monocle3 pseudotime analysis suggested that these identified different subclusters of iTrophoblast may also represent different transition states during iTrophoblast maturation (Figures 3N and 4Q; Figure S3P, Supporting Information). In normal placenta, the initial iTrophoblast subcluster iTrophoblast1 was eventually transformed into specific functional iTrophoblast4 through iTrophoblast2 and iTrophoblast3 subclusters. Whereas in PE placenta, there was an abnormal transformation branch, that is, iTrophoblast1 was converted into iTrophoblast2 and then transformed into abnormal iTrophoblast5, which was closer to NK and T cells (Figure 4R). In fact, by comparing the DEGs of normal and PE placenta in different iTrophoblast subclusters, we found that the difference of iTrophoblast1 in normal and PE placenta was not significant, and its features were closer to trophoblast cells (Figure 4R; Figure S5P, Supporting Information). iTrophoblast2 located in the center of iTrophoblast transformation branch was closer to HBC, decidual macrophages, and monocytes, which were significantly enriched in immune activation and differential multivesicular body‐lysosome fusion, suggesting that iTrophoblast may require the trophoblast‐like cells to transform into cells with phagocytic functions similar to macrophages, so that they could phagocytose other cells^[^ ^45^ ^]^ (Figure 4P,R; Figures S3Q, and S5O, Supporting Information). Suitable regulatory processes allowed iTrophoblast to be converted from iTrophoblast2 to iTrophoblast3, or otherwise to iTrophoblast5 in an abnormal state. Subsequently, we integrated and reclustered the iTrophoblast subclusters identified from human, mouse, and rat placentas, and found that iTrophoblasts of different species had both species‐specific and relatively conserved features (Figure S5Q,R, Supporting Information). In the reclustered subcluster4, iTrophoblast5 from human placenta with shallow trophoblast invasion characteristics and iTrophoblast2 and iTrophoblast3 from mouse were clustered together, which are the subclusters identified above that may lead to weaker trophoblast invasion compared with rat (Figure 3P). Furthermore, based on the different placental cell characteristics identified at the single‐cell level, a BayesPrism model was trained. We inferred the proportion scores of iTrophoblast subclusters from published bulk RNA‐seq of normal and PE placenta samples^[^ ^46^, ^47^ ^]^ and found that normal placenta samples had more mature iTrophoblast4 subclusters (Figure S5S,T, Supporting Information), showing diagnostic value for preeclampsia.

In summary, by using the preeclampsia disease model, we validated the regulatory mechanism of trophoblast invasion obtained from the above cross‐species comparison of rats and pigs, among which the most important iTrophoblast disorder may be the potential pathogenesis of preeclampsia.

### 2.5 Spatial Identification and Visualization of ITrophoblast in Normal and Preeclamptic Placentas Using ScStereo‐seq

**Figure 5:**
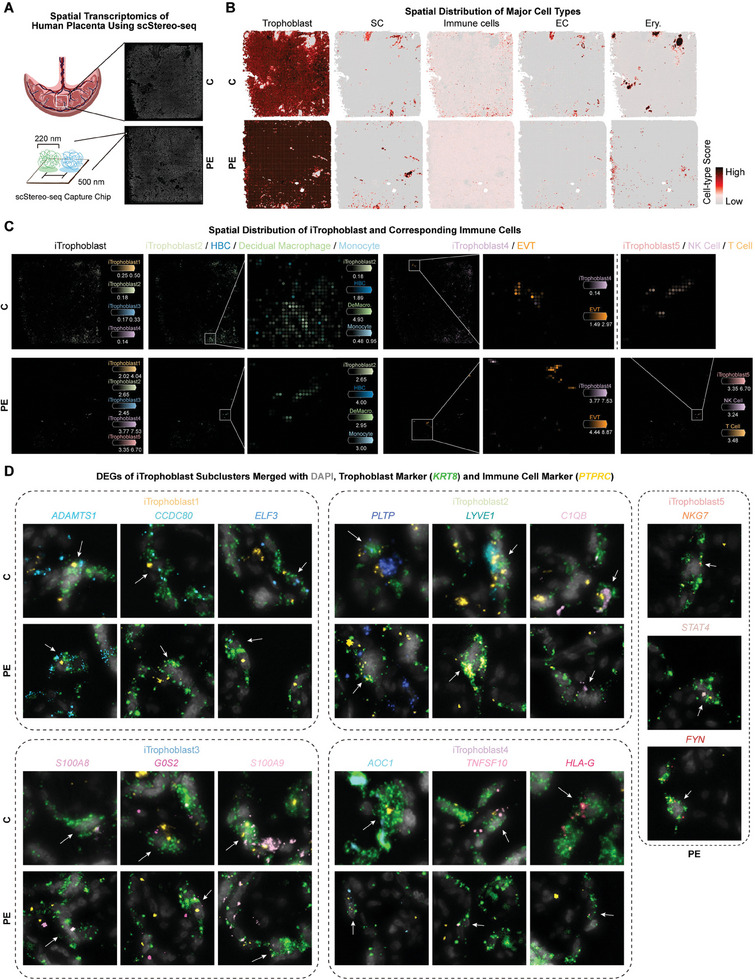
Spatial identification and visualization of iTrophoblast in normal and preeclamptic placentas using scStereo‐seq. A) Schematic diagram of placenta samples for spatial transcriptomics (scStereo‐seq). B) Spatial distribution of spots colored by placental major cell type scores in scStereo‐seq data. C) The identified cell subclusters from scRNA‐seq atlas mapped to scStereo‐seq profile by cell2location deconvolution. D) In situ hybridization of iTrophoblast subcluster DEGs merged with *KRT8* and *PTPRC*.

Nonetheless, a comprehensive insight into iTrophoblast cells in normal and preeclamptic placentas remains very difficult. Therefore, we performed scStereo‐seq^[^ ^48^ ^]^ on normal and PE placental tissue sections (**Figure** **5**A; Figure S8A, Supporting Information). We identified and located 5 major placental cell types in placental tissue sections (Figures 4B and 5B; Figure S8B, Supporting Information). Strikingly, the visualization of spatial distribution revealed abundant trophoblast cells with scattered immune cells, consistent with the physical layer observed in traditional hematoxylin and eosin (H&E) staining of placental tissue sections (Figure 5B).^[^ ^49^ ^]^

Remarkably, when visualizing different iTrophoblast subclusters in the placenta using scStereo‐seq, we observed that iTrophoblast1‐4 in normal placenta tissue and iTrophoblast1‐5 in PE placenta colocalized in spatial location, which to some extent shows a potential transformation of iTrophoblast subpopulations (Figure S8C, Supporting Information). Furthermore, the spatial colocalization of iTrophoblast2 with HBCs, decidual macrophages, and monocytes suggests a possible connection between iTrophoblast2 and macrophages (Figure 5C). The colocalization of specific functional iTrophoblast4 with EVT and iTrophoblast with EC suggested that it may play an indispensable role in the trophoblast invasion process (Figure 5B,C). Whereas the spatial colocalization of iTrophoblast5 and NK and T cells in PE placenta tissue revealed the abnormal transformation of iTrophoblast2 present in PE placenta (Figure 5C). In addition, in situ hybridization demonstrated the co‐expression of *KRT8*, *PTPRC*, and genes specific to different subclusters of iTrophoblast, which was consistent with the scStereo‐seq results (Figure 5D; Figure S8D, Supporting Information). These results provide precise and reliable verification of iTrophoblast subclusters in placental tissue.

### 2.6 Dysfunctional and Spatially Disorganized ITrophoblast Leads to Failure of Placental EVT Invasion and Preeclampsia

**Figure 6:**
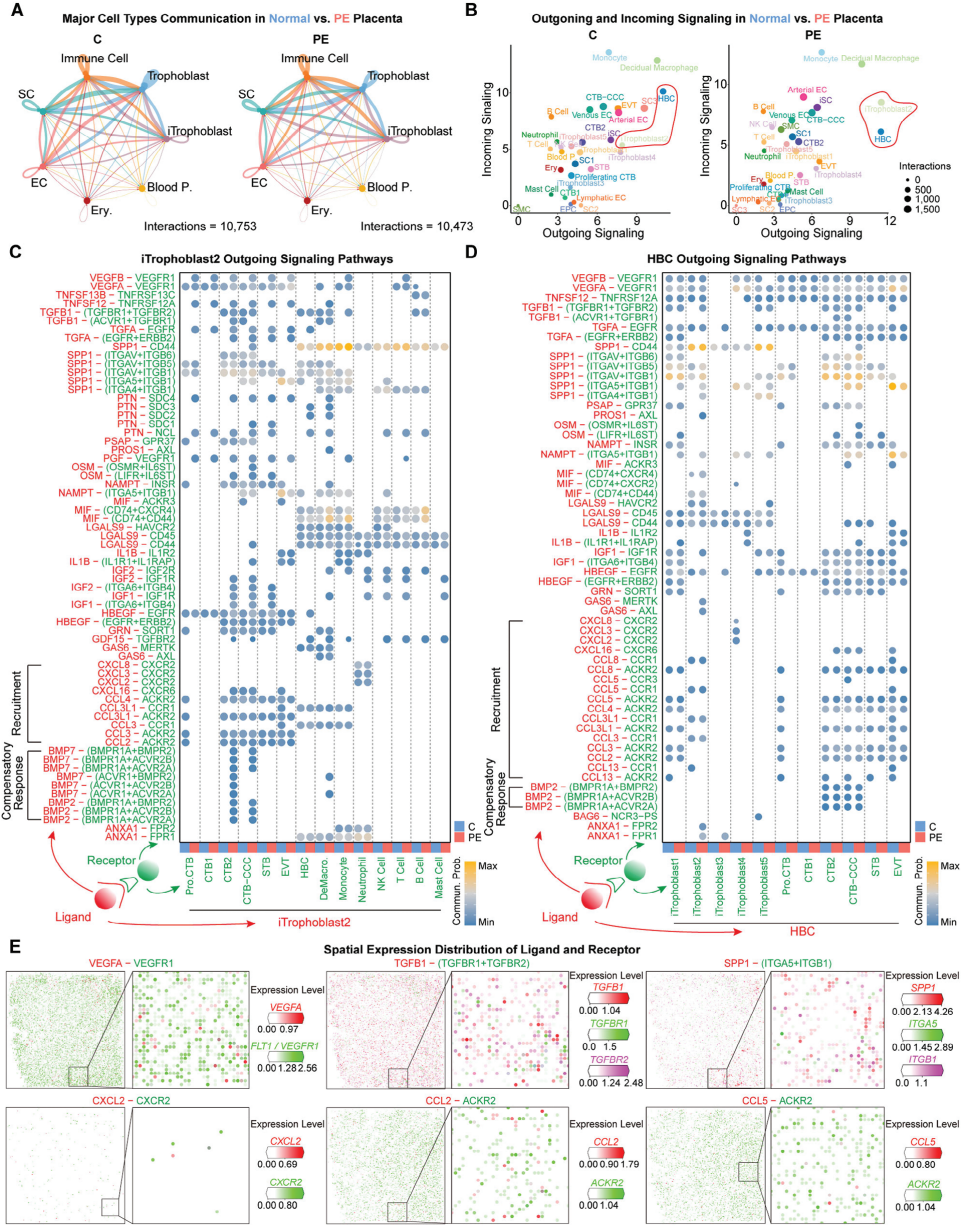
Dysfunctional and spatially disorganized iTrophoblast leads to failure of placental EVT invasion and preeclampsia. A) Network diagram for the number of ligand‐receptor pairs in main cell types between normal and PE placentas. Edge width is proportional to the number of cell interactions. B) Outgoing and incoming communication probabilities and number of interactions in different cell subclusters of placenta. C) Dot plot showing outgoing signals from the iTrophoblast2 subcluster emitted to trophoblast and immune cell subclusters. D) Dot plot showing outgoing signals from the HBC subcluster emitted to iTrophoblast and trophoblast subclusters. E) scStereo‐seq heatmaps of normal placenta showing spatial expression of selected ligands and receptors.

To molecularly understand the pathological effects of iTrophoblast on abnormal trophoblast invasion and preeclampsia, we paid special attention to the signaling pathway changes in PE placenta compared with normal placenta. We applied secreted signaling in CellChat to identify pathway communication between different types of placental cells.^[^ ^50^ ^]^ Then we found that ligand‐receptor interactions in PE placenta were significantly different compared to normal placenta (**Figure** **6**A,B; Figure S9A–C, Supporting Information). Notably, we found that the CXCL signaling was obviously higher in normal placenta than PE placenta, while the opposite was true for BMP signaling (Figure S9A–C, Supporting Information). This is consistent with previous studies that CXCL recruits immune cells to maintain a special immune microenvironment at the maternal‐fetal interface, while participating in angiogenesis and regulating trophoblast invasion.^[^ ^51^, ^52^ ^]^ Although BMP signaling has been shown to promote trophoblast invasion in vitro,^[^ ^53^ ^]^ recent studies have also reported a negative correlation with clinical PE samples consistent with our results, and its enhanced signaling could serve as a compensatory response for shallow trophoblast invasion in PE.^[^ ^54^ ^]^

Interestingly, when we compared the outgoing and incoming signaling of all placental cell subclusters in normal placenta and PE placenta, we found clear abnormalities in the interaction signaling of iTrophoblast2 and HBC, which is the turning point identified above during the process of iTrophoblast transformation (Figures 4N, and 6B). Next, we focused on the outgoing signaling of iTrophoblast2 toward trophoblast and immune cells and HBC toward iTrophoblast and trophoblast cells (Figure 6C,D). We found that the interaction between iTrophoblast2 and proliferative cytotrophoblast (CTB) through CCL2/3/4 was missing in the PE placenta, while its interaction with CTB2 and cytotrophoblast cell column‐cytotrophoblast (CTB‐CCC) through BMP2/7 was unique in PE (Figure 6C). It is worth noting that among the outgoing signaling of HBC, its interaction with proliferative CTB through CCL2/3/4/5/8/13 was still missing in PE, whereas the interaction with functional iTrophoblast4 through CXCL2/3/8 was also unique in normal placenta (Figure 6D). Therefore, we also focused on the outgoing signaling of functional iTrophoblast4 toward trophoblast and immune cells. To our surprise, almost all interactions between iTrophoblast4 and EVT were missing in PE placenta (Figure S9D, Supporting Information), including ANGPTL4 signaling, which is important for angiogenesis regulation during trophoblast invasion.^[^ ^55^, ^56^ ^]^ Furthermore, spatial colocalization of ligand and receptor expression demonstrated signaling communication by these cells in placenta tissue (Figure 6E; Figure S9E, Supporting Information).

In summary, we conclude that dysfunctional and spatially disorganized iTrophoblast may disrupt the EVT invasion process during human placentation and may ultimately lead to EVT invasion failure and preeclampsia.

## 3 Discussion

As the first organ formed in mammals, the placenta at maternal‐fetal interface is the basis of maternal pregnancy and fetal development.^[^ ^6^ ^]^ However, due to ethical restrictions, research on placentation is destined to not be directly implemented in humans. Therefore, in this study, we used a rat model that is closer to humans, a universal mouse model, and a relatively different pig model to analyze the regulation of placentation and trophoblast invasion at single‐cell level. Then, we used the human preeclampsia model to validate the regulatory mechanisms of trophoblast invasion identified across species and to deepen our understanding of preeclampsia pathology.

Overall, by leveraging the strengths and weaknesses between species, we demonstrated that a detailed understanding of placentation can be obtained through comparisons between rats and pigs, not only species‐specific but also common across species. We identified some important functional gene modules in the process of trophoblast invasion which were verified in PE placenta with shallow trophoblast invasion. Similarly, previous studies have confirmed changes in actin cytoskeletal organization during trophoblast invasion,^[^ ^57^, ^58^ ^]^ and recent studies have also demonstrated the crucial roles of extracellular vesicle‐related crosstalk in trophoblast invasion and pregnancy disorders.^[^ ^59^, ^60^ ^]^ Due to the non‐invasive feature of pig epitheliochorial placenta, the pig placenta in early pregnancy is almost independent of the mother.^[^ ^13^ ^]^ The large number of HBCs identified in an independent placental hematopoietic system again emphasized the de novo generation of macrophages in placenta.^[^ ^61^ ^]^

Remarkably, we have identified a dual‐characteristic type of iTrophoblast with trophoblast and immune‐related features in the invasive placenta. According to the different developmental states, these iTrophoblast could be divided into immature iTrophoblast and mature iTrophoblast. Although iTrophoblast in rat and human placentas were identified as distinct iTrophoblast subclusters, these iTrophoblast subclusters all seemed to gradually transform into the final iTrophoblast subcluster with regulatory roles for trophoblast invasion. For example, iTrophoblast4 in normal human placenta had similar EVT characteristics but is not EVT. It can interact with EVT through ANGPTL4, which plays an important role in trophoblast invasion and angiogenesis.^[^ ^55^, ^56^ ^]^ The transformation disorder of iTrophoblast produced an abnormal iTrophoblast subcluster, resulting a large number of immature iTrophoblast in PE placenta. This disrupted the trophoblast invasion during placentation, eventually leading to the development of preeclampsia.^[^ ^62^ ^]^

Recently, an increasing number of non‐immune cells with immune features have been reported in diverse biological contexts.^[^ ^21^, ^63^ ^]^ These cells with dual characteristics exist in a variety of complex microenvironments, including tumors, decidua, and the placenta. For example, at the mouse implantation site, Yang et al.^[^ ^21^ ^]^ discovered that immune‐featured stromal cells recruit and suppress immune cells and govern vascularization to establish decidual homeostasis. Although we also found iSCs in the placenta, their regulatory role in trophoblast invasion may not be as specific as iTrophoblast because they exist in both invasive and non‐invasive placentas. Interestingly, we found that these iSCs, like iTrophoblast, could also be divided into immature and mature states. Based on our description of iTrophoblast, this leads us to speculate whether the abnormal accumulation of these immature non‐immune cells with immune features contributes to the progression of some diseases including preeclampsia, but this still requires further exploration.

To confirm iTrophoblasts roles in placental tissue, we tried to isolate iTrophoblasts from placental tissue using flow cytometry, but trophoblast markers such as KRT7 and KRT8 are intracellular proteins, it is not possible to sort out live iTrophoblasts for further experimental verification. A dedicated iTrophoblast transgenic mouse model is essential for studying the significance of this cell type in placental development. In this study, we used immunofluorescence staining to confirm iTrophoblasts in the placental villi at the maternal‐fetal interface during the first trimester of human pregnancy, and immunofluorescence staining of additional decidua helped to support the statement of iTrophoblast functions.

In conclusion, this study provides a better understanding of the complex events during placentation. Cross‐species comparisons suggest potential regulation of trophoblast invasion by iTrophoblast during placentation and the progression of preeclampsia due to abnormal iTrophoblast transformation. This study aims to establish a complementary approach to deepen our understanding of development and provide a good stepping stone for further research into improving related pregnancy complications.

## 4 Experimental Section

### Animals

The animal experiments and procedures involved in this study were approved by the Ethics Committee of the Laboratory Animal Center of South China Agricultural University (permit number: SYXK‐2022‐0136).

Specific pathogen‐free (SPF) Sprague‐Dawley female and male rats (8–10 weeks old) were purchased from Liaoning Changsheng Biotechnology Co., Ltd. Rats were fed regular formula feeds and housed in a controlled room at 22 °C with a 12:12 h light‐dark cycle. Then, the rats were used for timed mating, with the morning of vaginal plug appearance recorded as embryonic day 0.5 (E0.5). The female SD rats with vaginal plugs were sacrificed by cervical dislocation at the following pregnancy time points: E9.5, E12.5, and E15.5, and placental samples were collected from the uterine horns on both sides. Some were used to dissociate the tissue and obtain single‐cell suspension, and the remainder were fixed in 4% paraformaldehyde for 24 h, embedded in paraffin, and sectioned for immunofluorescence.

For pigs, healthy and disease‐free sows from a local commercial farm were checked for estrus twice daily and artificially inseminated after estrus with a standard dose of single semen. The day of artificial insemination was marked as day 0 of pregnancy (P0). Sows were slaughtered at pregnancy time points of P16, P20, P24, and P28, and placental samples were also collected from bilateral uterine horns for tissue dissociation.

### Human Placental Samples

The collection of human placental samples involved in this study was approved by the Medical Ethical Committee of The First Affiliated Hospital, Sun Yat‐sen University (permit number: 2021–649), and all pregnant women provided written informed consent before the operation. Referring to the clinical standards of preeclampsia,^[^ ^44^ ^]^ normal (C) and preeclamptic (PE) placental samples were collected from an area ≈5 cm away from the central umbilical artery and 1–2 cm deep, while avoiding areas of infarction, hemorrhage, and calcification (Table S7, Supporting Information). The collected placenta tissue, similar to that of rat and pig placenta samples, some were used to dissociate and obtain the single‐cell suspension, some were used for OCT embedding, and the remainder were fixed with 4% paraformaldehyde and embedded in paraffin. Early pregnancy maternal‐fetal interface tissues were obtained for immunofluorescence analysis from elective terminations of normal pregnancies at 8 weeks gestation.

### Placental Tissue Dissociation and Single‐Cell Suspension Preparation

Placenta samples were minced into small pieces of ≈0.5 mm^3^ using surgical scissors. The cells were then dissociated using 0.2 mg ml^−1^ Liberase TL solution (Sigma) at 37 °C for 15 min. The dissociated cells were filtered through a 40 µm cell strainer (Corning) and centrifuged at 300 g for 5 min at 4 °C. After removing the supernatant, the pelleted cells were suspended in red blood cell lysis buffer (MACS) for 10 min at 4 °C to exclude mature red blood cells. Finally, cells were washed twice with ice‐cold PBS and resuspended in PBS supplemented with 0.04% BSA.

### Single‐Cell RNA‐Seq Library Generation and Sequencing

The concentration of single‐cell suspensions was adjusted to 700–1200 cells µl^−1^. Then, single‐cell suspensions were loaded on a Chromium Single Cell Controller (10X Genomics) according to the manufacturer's instructions (Chromium Next GEM Single Cell 3′ Reagent Kit V3.1). All subsequent steps were performed following the standard manufacturer's protocols. Purified libraries were analyzed by the Illumina NovaSeq 6000 platform using a paired‐end 150‐bp sequencing strategy.

### Data Processing of Single‐Cell RNA‐Seq Data

The 10X Genomics scRNA‐seq raw data were processed using the Cell Ranger pipeline (v.7.0.1) with the parameter “–include‐introns = False” to quantify gene counts. Rat and pig reference genome and gene annotation files were downloaded from the Ensembl website and indexed using the mkref function. The human reference genome and gene annotation files were obtained directly from the 10X Genomics website. The mouse data was obtained from a previous study.^[^ ^16^ ^]^ Further downstream analyses were performed in the R package Seurat (v.5.0.0).^[^ ^64^ ^]^

### Quality Control of ScRNA‐Seq Data

Strict quality filtering criteria were performed to filter out low‐quality cells based on three metrics: number of detected genes, unique molecular identifier (UMI) counts, and percentage of mitochondrial gene expression. Specifically, cells expressing at least 500 genes and with a mitochondrial gene count below 15% were used for follow‐up analysis. Only genes expressed by more than three cells were retained for subsequent analysis.

### Dimension Reduction and Unsupervised Clustering

Highly variable genes (HVG) were calculated by the FindVariableFeatures function, using the parameter “nfeature = 5000”. Then, the IntegrateLayers function in Seurat5.0 was used to integrate datasets of samples at different stages through the RPCAIntegration method. Rpca (1–40) reduction was selected to perform the RunUMAP function and cell clusters were identified through a shared nearest neighbor (SNN) modularity optimization‐based clustering with FindClusters function. The major cell types were first annotated in the placenta of each species. Then, the subset function wad used to re‐cluster trophoblast, SC, immune cells, and EC, repeated the above steps to obtain subclusters.

### Cell Doublet Detection

Artifacts generated from multiple cells were detected by applying the Scrublet method^[^ ^20^ ^]^ with a standardized approach to all data. Referring to the limitations of 10X Genomics single‐cell sequencing technology, it was assumed that 7% doublets were present in each sample and used the parameter “expected_doublet_rate = 0.07” to execute the Scrublet function to detect doublets.

### Differentially Expressed Gene Identification

The differentially expressed genes between different clusters were identified using the FindAllMarkers function based on the Wilcoxon rank sum test with parameters “logfc.threshold = 0.5, min.pct = 0.5, only.pos = TRUE”, and genes with corrected p‐value < 0.05 were selected as DEGs.

### GO Enrichment Analysis

GO enrichment analysis of genes was implemented by the R package clusterProfiler (v.4.6.2)^[^ ^65^ ^]^ and visualized by ggplot2 (v.3.4.1). GO terms with corrected FDR values < 0.05 were defined as significantly enriched. For enriched GO terms in SC subclusters, the EnrichmentMap and AutoAnnotate apps in Cytoscape (v.3.9.1) were used to cluster the terms of each subcluster and to generally annotate ontology terms for the cluster.

### Pseudotime Trajectory Inference

The monocle3 (v.1.3.1) and monocle2 (v.2.22.0)^[^ ^66^ ^]^ algorithms were used to construct single‐cell pseudotime trajectories to discover changes in iTrophoblast cells, respectively. Monocle3 was used to highlight pseudotime changes in cell subclusters, whereas monocle2 to show changes in cellular development over stages. Additionally, the R package CytoTRACE (v.0.3.3)^[^ ^67^ ^]^ was used to identify differences in transcriptional diversity during cell development, thereby calculating the CytoTRACE score for each cell to predict differentiation status. CytoTRACE scores range from 0 to 1, with higher scores indicating less differentiation.

### Identification of Auto‐Correlated Gene Modules

Modules of correlated genes were identified using the WGCNA method.^[^ ^68^ ^]^ The expression matrices of conserved genes with comparable cells from rats and pigs were used as input. An appropriate soft‐thresholding value β was calculated to establish the scale‐free topology model using the “pickSoftThreshold” function. Topological overlap matrix with parameters (minModuleSize = 30 and mergeCutHeight = 0.25) was generated to quantify similarity, followed by dynamic tree cutting to identify gene modules.

### Protein‐Protein Interaction (PPI) Network Analysis of M1

The STRING database (https://string‐db.org) was applied to obtain potential interactions between genes in identified invasive trophoblast M1. The PPI network with higher confidence scores ≥ 0.9 were visualized through Cytoscape and hub modules were identified using the MCODE app.

### ScStereo‐Seq Library Preparation and Sequencing

The sample library of scStereo‐seq was constructed according to the STOmics Gene Expression Kit standard protocol V1.2 as described previously.^[^ ^48^ ^]^ Frozen tissue sections of ≈10 mm were adhered to the scStereo‐seq capture chip (spot size 220 nm and center‐to‐center distance 500 nm), incubated and fixed in −20 °C methanol for 30 min, and then stained with nucleic acid dyes and imaged. For permeabilization, placental tissue sections attached to the chip were permeabilized at 37 °C for 12 min. The RNA released in situ was then captured by DNA nanospheres (DNBs) on the chip, and reverse transcribed to generate cDNA, which was amplified and purified. Finally, DNBs were loaded into the patterned Nano arrays and sequenced on MGI DNBSEQ‐T7 sequencer.

### Data Processing of ScStereo‐Seq Data

Paired‐end raw reads generated from the MGI DNBSEQ‐T7 sequencer were processed using BGI Stereomics' standard analysis pipeline.^[^ ^48^ ^]^ To fully characterize the spatial transcriptomic landscape of placenta, a bin size of 100 (100 × 100 DNB) was used as the fundamental analysis unit (spot) for downstream analyses. Then, the SCTransform function was used to normalize and scale the raw gene expression matrix of scStereo‐seq data.

### Cell Type Annotation of ScStereo‐Seq Data

Spots were annotated according to the reference‐based integration workflow in Seurat. Briefly, the FindTransferAnchors and TransferData functions in Seurat were used to obtain a probabilistic classification of major cell types identified in the placental scRNA‐seq data on scStereo‐seq data. To further annotate all cell subclusters in scRNA‐seq, a second round of annotation was performed by deconvolution analysis of spots through the cell2location algorithm.^[^ ^69^ ^]^

### Cell‐Cell Communication Analysis

Interactions between each cell subcluster in the placenta were inferred by using the human‐secreted signaling database and standard workflows in CellChat (v.1.6.1).^[^ ^50^ ^]^

### Deconvolution of Different ITrophoblast Subclusters from Bulk RNA‐Seq Data

Deconvolution of iTrophoblast subclusters was implemented using the BayesPrism algorithm.^[^ ^70^ ^]^ Briefly, placental scRNA‐seq data was used and constructed a reference matrix based on the characteristics of different cell types to deconvolute the cellular abundance of iTrophoblast subclusters in normal and PE placenta bulk RNA‐seq. The bulk RNA‐seq data were obtained from GSE203507^[^ ^46^ ^]^ and GSE148241^[^ ^47^ ^]^ (Table S8, Supporting Information). Default parameters were used for the deconvolution analysis.

### Flow Cytometry

Single‐cell suspensions were prepared from placentas as described above. To detect the cell surface marker CD45, single‐cell suspensions were stained with Alexa Fluor 488‐conjugated anti‐CD45 (Abcam, ab315958, 1:500 dilution) for 30 min at 4 °C. For the intercellular marker KRT7, single‐cell suspensions were subsequently fixed, permeabilized, and blocked before incubation with Alexa Fluor 647‐conjugated anti‐KRT7 (Abcam, ab192077, 1:500 dilution). Isotype and single‐staining controls were included. Stained cells were examined using a CytoFlex (Beckman) flow cytometer. All raw data were analyzed using FlowJo software (Tree Star Inc.).

### Immunofluorescence Staining

Four micron‐thick sections from 4% paraformaldehyde‐fixed and paraffin‐embedded placental tissue were dewaxed and hydrated, antigen retrieval was performed with sodium citrate antigen retrieval buffer (pH 6.0), and endogenous peroxides were quenched with 3% H_2_O_2_. Then, sections were blocked with 3% BSA for 30 min at room temperature and incubated with the primary antibody overnight at 4 °C followed by the corresponding secondary antibody for 50 min at room temperature. After the first primary antibody incubation was completed, the primary and secondary antibodies that had been bound to the tissue were removed using sodium citrate antigen retrieval buffer, and the second primary antibody was incubated in the same manner. Finally, the nuclei were counterstained with DAPI, and the sections were observed under a microscope.

### In Situ RNA Hybridization

The specific probe pairs for target RNA were designed by Spatial FISH Ltd. (Shenzhen, China). Specifically, paraffin tissue sections were dehydrated and denatured with methanol and then incubated with the specific targeting probe pairs in hybridization buffer (10% deionized formamide, 2X SSC, 10% dextran sulfate, and 2 mm VRC) at 37 °C overnight. Sections were washed 3 times with PBST and incubated with target probes in a ligation mixture at 25 °C for 3 h. The sections were washed thrice with PBST again and subjected to rolling circle amplification by Phi29 DNA polymerase at 30 °C overnight. Subsequently, the fluorescent detection probes were incubated with sections for the single‐molecule detection of specific RNA. The sections were imaged using Leica TCS SP8 and Olympus FV3000 confocal microscopes.

### Statistical Analysis

All statistical analyses were performed using R (v.4.2.3). The two‐tailed Student's t test was used to determine the significance of the difference between two groups, and *P* < 0.05 was considered statistically significant. The Wilcoxon rank sum test was used to determine the differentially expressed genes between different cell types, the *P* values were subsequently corrected using the Benjamini‐Hochberg method, and the corrected *P* < 0.05 was considered statistically significant.

## Conflict of Interest

The authors declare no conflict of interest.

## Author Contributions

X.Z. and D.Z. contributed equally to this work. T.G., G.C., and L.H. conceived the project. X.Z. performed all the bioinformatic analyses. X.Z. and D.Z. drafted the manuscript. W.W. conceived the figure layout and drew the schematics. X.Z., W.W., Y.D., Y.W., S.G., Y.S., J.G., and L.J. prepared samples and performed the experiments. D.Z. and F.M. contributed to the collection of samples. J.S., Z.X., S.H., Z.L., Z.W., T.G., G.C., and L.H. revised the manuscript. All authors read and approved the final version of the manuscript.

## Supporting information

Supporting Information

Supplemental Table 1

Supplemental Table 2

Supplemental Table 3

Supplemental Table 4

Supplemental Table 5

Supplemental Table 6

Supplemental Table 7

Supplemental Table 8

## Data Availability

The scRNA‐seq data generated in this study are deposited in the Genome Sequence Archive of the National Genomics Data Center (NGDC) under the accession numbers CRA014556 and CRA014591.
