# Supplementary material for: Cross‐Species Insights into Trophoblast Invasion During Placentation Governed by Immune‐Featured Trophoblast Cells
Source: Adv Sci (Weinh). 2024 Sep 5;11(42):2407221. doi: 10.1002/advs.202407221 (PMC11558115; doi:10.1002/advs.202407221)
Supplement: Supplementary file 1 — Supporting Information [file ADVS-11-2407221-s006.docx]

Supporting Information

Cross-species Insights into Trophoblast Invasion during Placentation Governed by Immune-featured Trophoblast Cells

Xupeng Zang, Dan Zhang, Wenjing Wang, Yue Ding, Yongzhong Wang, Shengchen Gu, Yijun Shang, Jianyu Gan, Lei Jiang, Fanming Meng, Junsong Shi, Zheng Xu, Sixiu Huang, Zicong Li, Zhenfang Wu, Ting Gu,* Gengyuan Cai* and Linjun Hong*


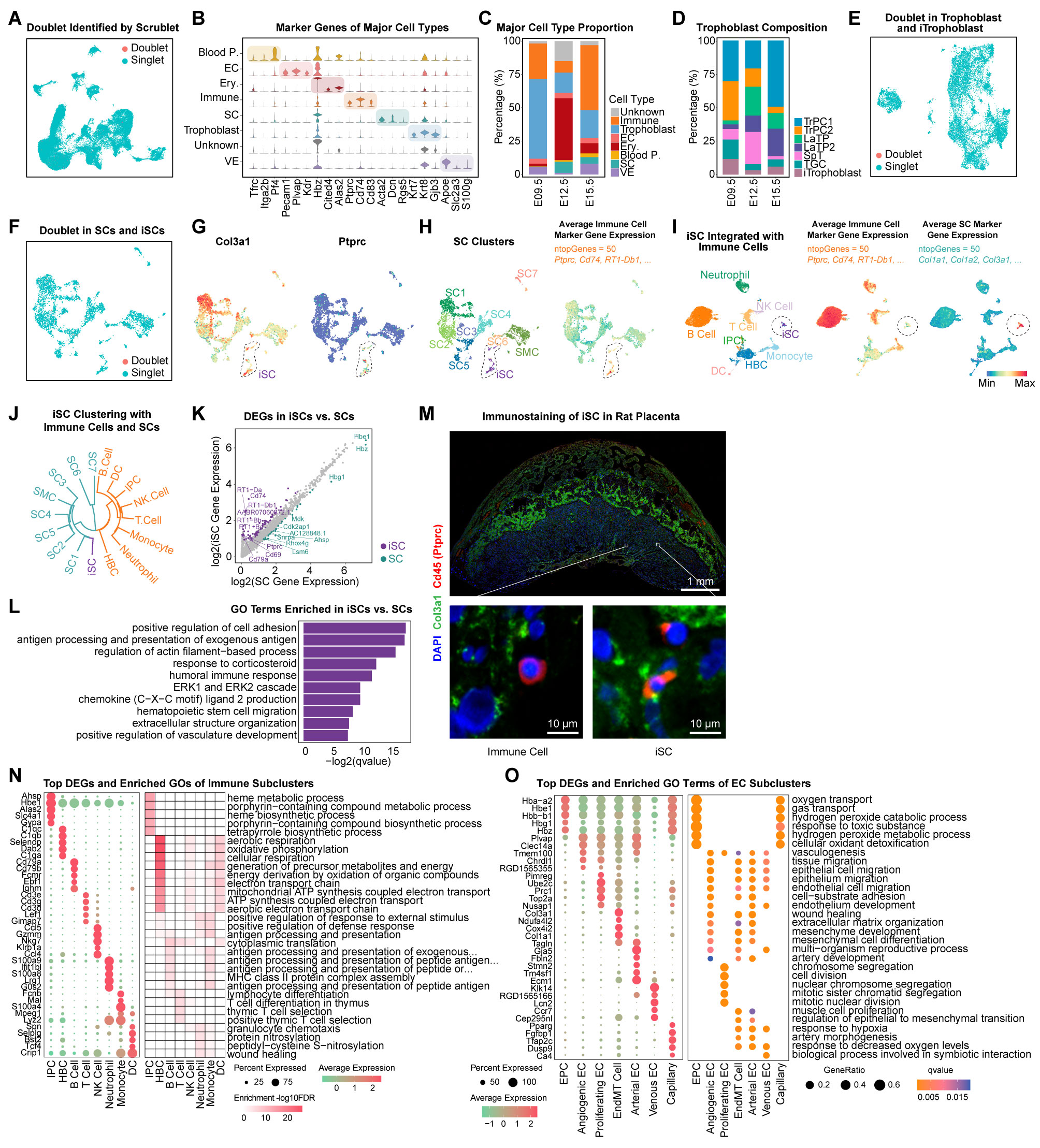


**Figure S1.** Decoding single-cell transcriptome profiles during rat placentation using scRNA-seq. A) UMAP visualization showing predicted doublets from rat placental single-cell transcriptome data using Scrublet. B) Violin plot showing expression of classical marker genes in major cell types. C) Stacked bar plot showing the proportion of major cell types in three stages. D) Stacked bar plot showing the proportion of each trophoblast subcluster in three stages. E) UMAP visualization showing predictions of singlet and doublet cells in iTrophoblast and trophoblast subclusters. F) UMAP visualization showing predictions of singlet and doublet cells in iSC and SC subclusters. G) UMAP visualization showing typical maker gene expression of stromal (*Col3a1*) and immune (*Ptprc*) cell in clustered SC subclusters. H and I) UMAP visualization showing the results of iSC integration with SC and immune cell subclusters, and the corresponding average expression levels of SC and immune cell marker genes. J) Hierarchical clustering of iSC, SC and immune cell subclusters. K) Scatter plot showing DEGs of iSCs compared to SCs. L) GO enrichment terms of DEGs in iSCs compared with SCs. M) Immunostainings of Col3a1 and Cd45 (Ptprc) protein in E15.5 rat placenta. N) Dot plot showing the top DEGs in immune cell subclusters, and the heatmap showing the enriched GO terms. O) Dot plot showing the top DEGs in EC subclusters and the enriched GO terms.


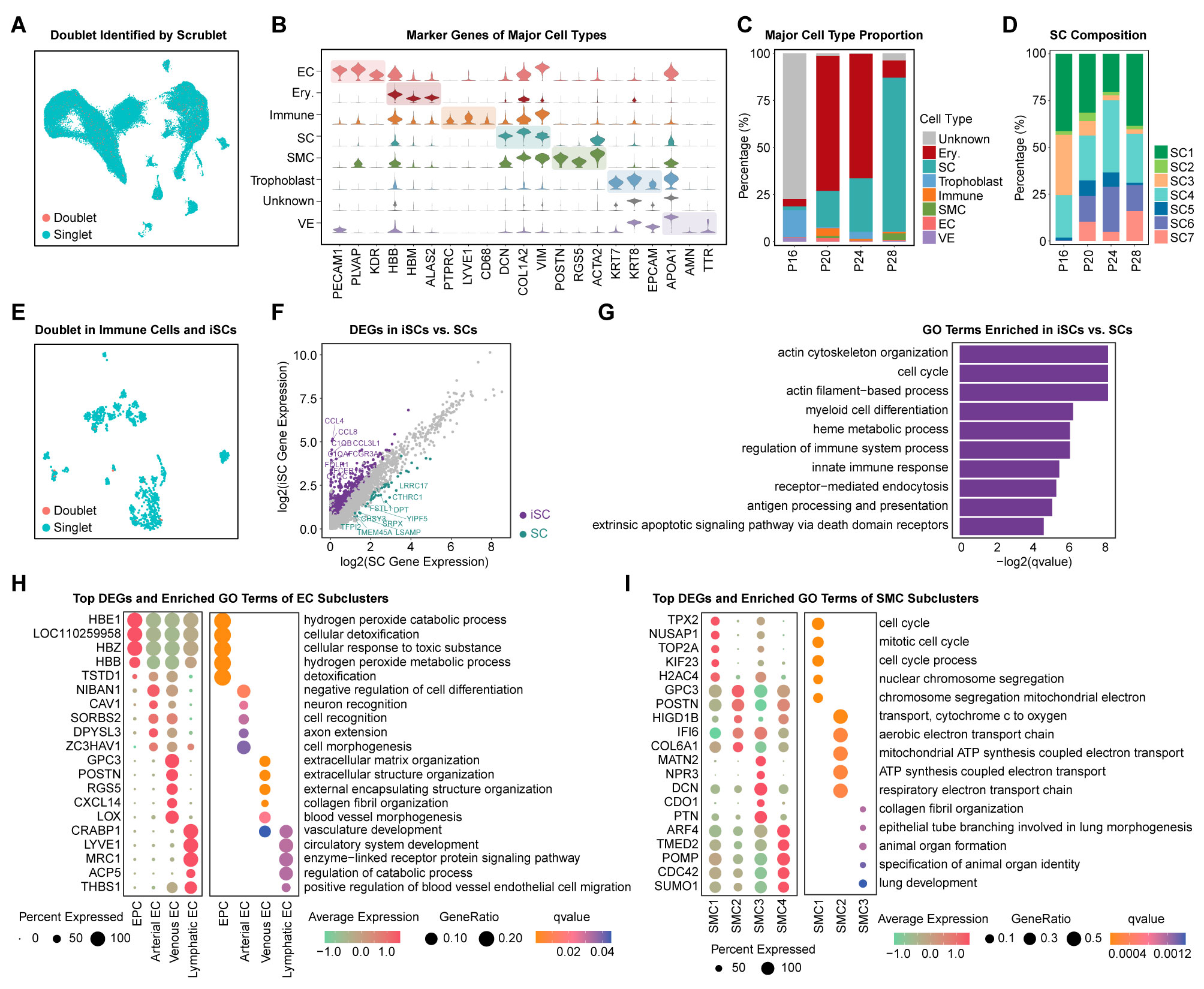


**Figure S2.** Constructing of the single-cell transcriptome atlas during early placentation in pigs. A) UMAP visualization showing predicted doublets from pig placental single-cell transcriptome data using Scrublet. B) Violin plot showing expression of classical marker genes in major cell types. C) Stacked bar plot showing the proportion of major cell types in four stages. D) Stacked bar plot showing the proportion of each SC subcluster in four stages. E) UMAP visualization showing predictions of singlet and doublet cells in iSC and immune cell subclusters. F) Scatter plot showing DEGs of iSCs compared to SCs. G) GO enrichment terms of DEGs in iSCs compared with SCs. H) Dot plot showing the top DEGs in EC subclusters and the enriched GO terms. I) Dot plot showing the top DEGs in SMC subclusters and the enriched GO terms.


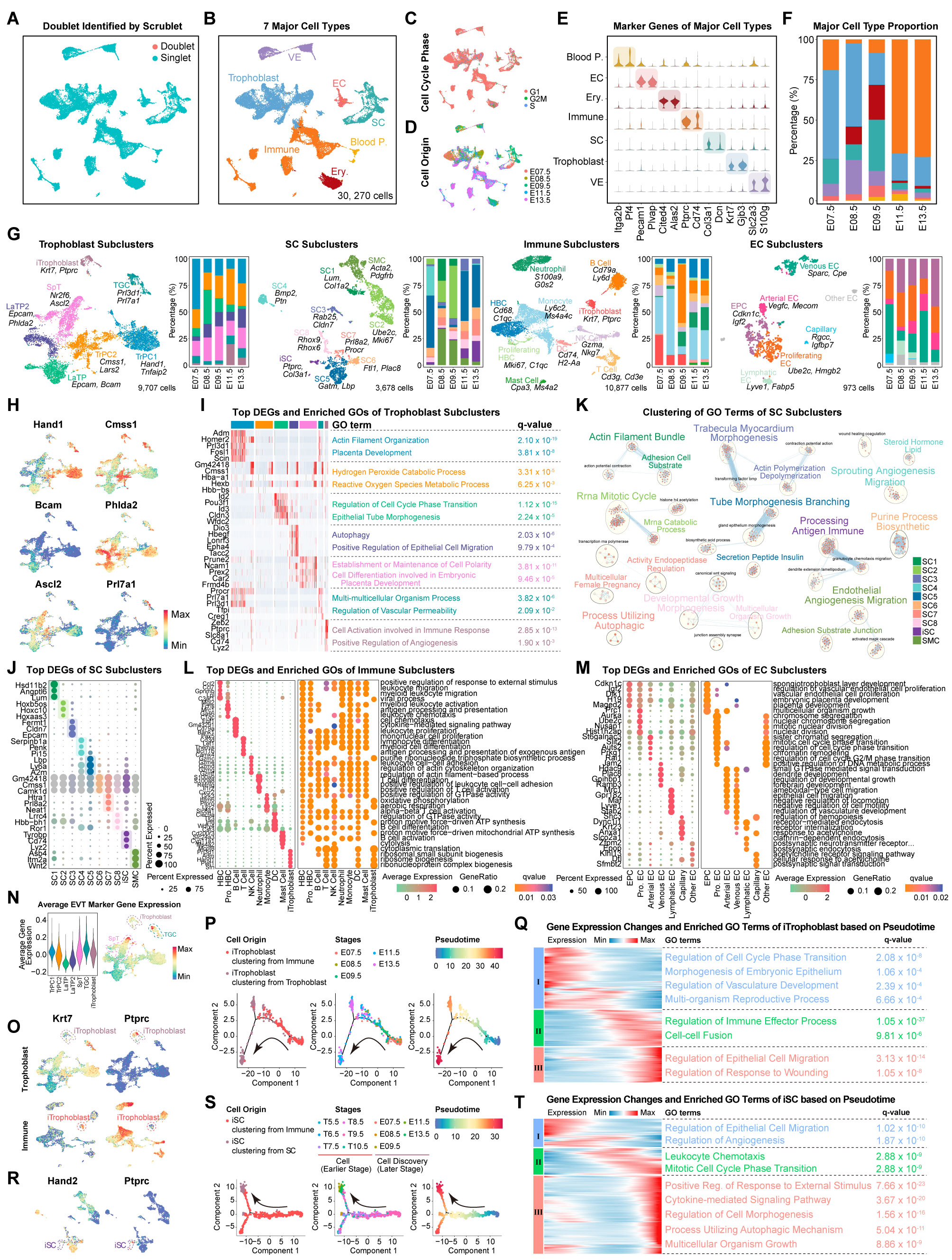


**Figure S3.** Reconstruction of single-cell transcriptome profiles during mouse placentation.^[1]^ A) UMAP visualization showing predicted doublets from mouse placental single-cell transcriptome data using Scrublet. B) UMAP visualization showing seven major cell types. C) UMAP visualization showing the cell cycle phase of mouse placental cells. D) UMAP visualization showing the origin of placental cells at different stages. E) Violin plot showing expression of classical marker genes in major cell types. F) Stacked bar plot showing the proportion of major cell types in five stages. G) UMAP visualization showing subclusters of mouse placental cell types, with a stacked bar plot showing the proportion corresponding to each subcluster. H) UMAP visualization showing marker gene expression in different placental trophoblast subclusters. I) Heatmap showing the top DEGs of trophoblast subclusters, and the GO biological process terms enriched by corresponding subcluster DEGs. J) Dot plot showing the expression of top DEGs in SC subclusters. K) GO term clustering results of SC subclusters. L) Dot plot showing the top DEGs in immune cell subclusters and the enriched GO terms. M) Dot plot showing the top DEGs in EC subclusters and the enriched GO terms. N) Violin plot and UMAP visualization showing the average expression levels of top 50 EVT marker genes in mouse different trophoblast subclusters. O) UMAP visualization showing typical marker gene expression of trophoblast (*Krt7*) and immune (*Ptprc*) cells in clustered trophoblast and immune cell subclusters. P) Predicted trajectories of iTrophoblast colored by cell origin, stage, and pseudotime. Q) Heatmap showing the clustering of DEGs along iTrophoblast trajectory, and corresponding representative GO terms. R) UMAP visualization showing typical maker gene expression of stromal (*Hand2*) and immune (*Ptprc*) cell in clustered SC subclusters. S) Predicted trajectories of iSC colored by cell origin, stage, and pseudotime. T) Heatmap showing the clustering of DEGs along iSC trajectory, and corresponding representative GO terms.


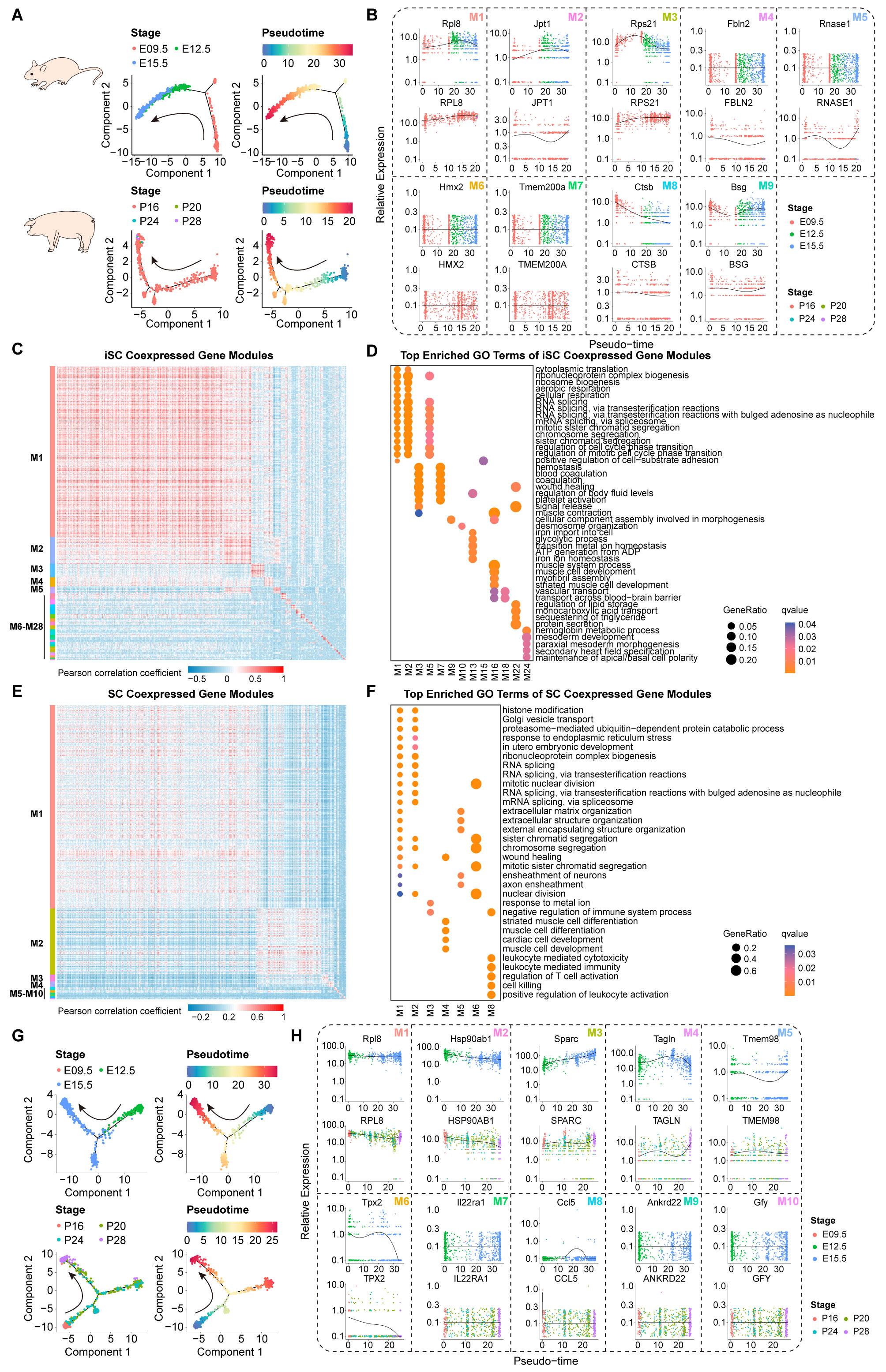


**Figure S4.** Cross-species comparative analysis of placental cells during rat and pig placentation. A) The upper layer showing predicted trajectories of TGCs in rats colored by stage and pseudotime. The lower showing predicted trajectories of Tr1 in pigs colored by stage and pseudotime. B) Expression changes of representative genes in invasive trophoblast coexpression modules along developmental trajectories. C) Heatmap showing correlation of coexpressed gene modules in iSC generated by WGCNA in rat and pig placentas. D) Dot plot showing the top enriched GO terms in iSC coexpressed gene modules. E) Heatmap showing correlation of coexpressed gene modules in selected SC generated by WGCNA in rat and pig placentas. F) Dot plot showing the top enriched GO terms in selected SC coexpressed gene modules. G) The upper layer showing predicted trajectories of SC1 in rats colored by stage and pseudotime. The lower showing predicted trajectories of SC3 in pigs colored by stage and pseudotime. H) Expression changes of representative genes in selected SC coexpression modules along developmental trajectories.


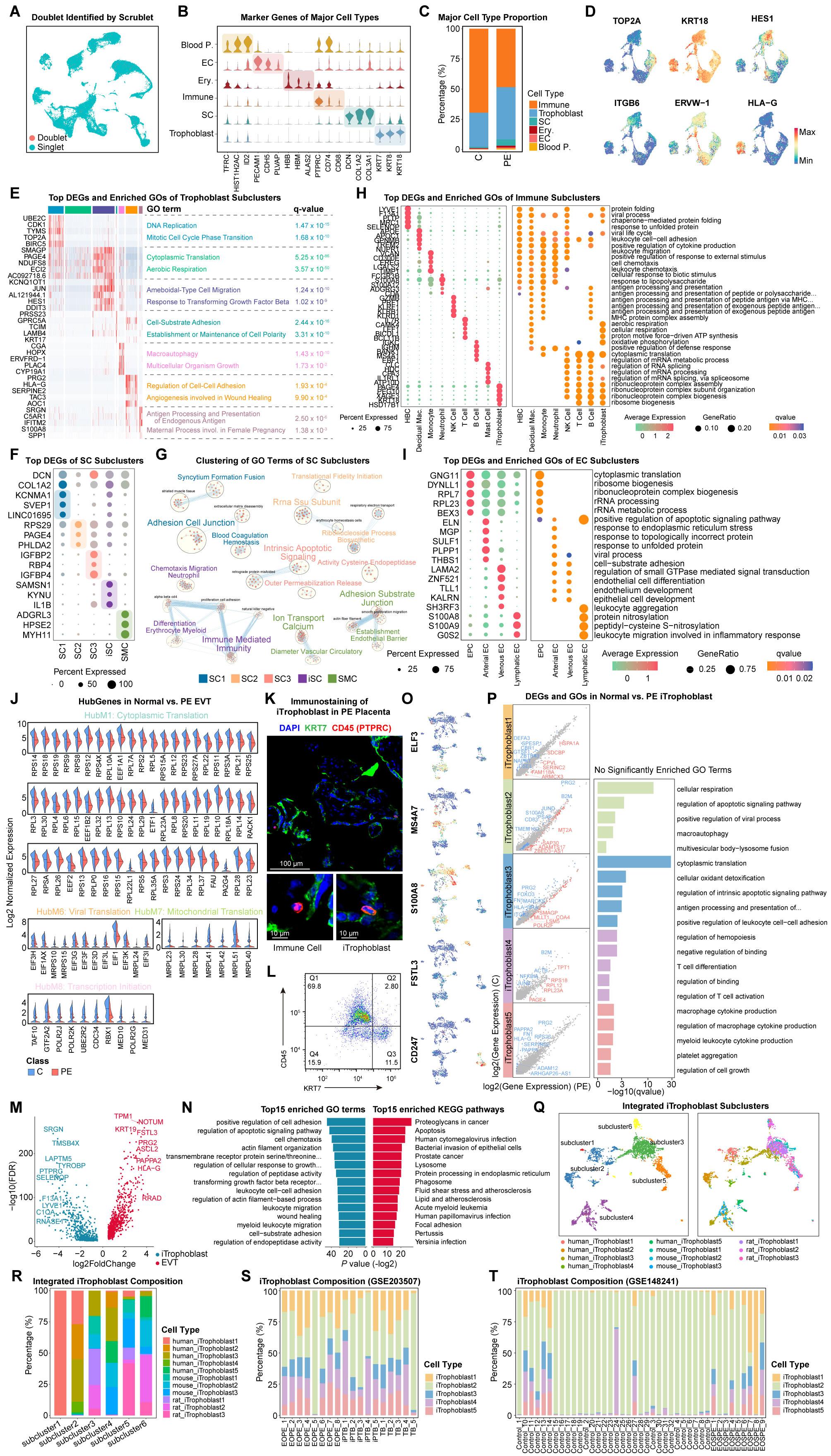


**Figure S5.** Functional cellular disorders regulating trophoblast invasion of placenta in human preeclampsia. A) UMAP visualization showing predicted doublets from human placental single-cell transcriptome data using Scrublet. B) Violin plot showing expression of classical marker genes in major cell types. C) Stacked bar plot showing the proportion of major cell types in two types of samples. D) UMAP visualization showing marker gene expression in different placental trophoblast subclusters. E) Heatmap showing the top DEGs of trophoblast subclusters, and the GO biological process terms enriched by corresponding subcluster DEGs. F) Dot plot showing the expression of top DEGs in SC subclusters. G) GO term clustering results of SC subclusters. H) Dot plot showing the top DEGs in immune cell subclusters and the enriched GO terms. I) Dot plot showing the top DEGs in EC subclusters and the enriched GO terms. J) Violin plots showing gene expression of hub modules 1 and 6-8 identified above in normal and PE placental EVT. K) Immunostainings of KRT7 and CD45 (PTPRC) protein in human PE placenta. L) Flow cytometry analysis of iTrophoblast expressing KRT7 and CD45 (PTPRC) in normal human placenta. M) Volcano plots showing DEGs between mature iTrophoblast and EVT subclusters. N) GO enrichment and KEGG pathway analysis of DEGs between mature iTrophoblast and EVT subclusters, showing the top 15 enriched terms for each category. O) UMAP visualization showing marker gene expression in different iTrophoblast subclusters. P) Scatter plot showing DEGs in each iTrophoblast subcluster in PE placenta compared with the normal placenta. Q) UMAP visualization showing clustering of iTrophoblast subclusters after integration of human, mouse, and rat iTrophoblasts. R) Stacked bar plot showing the origin of iTrophoblast subclusters after clustering. S) Stacked bar plot showing the iTrophoblast subclusters for each sample in the GSE203507 dataset^[2]^ deconvolution by BayesPrism. EOPE, early-onset preeclampsia; iPTB, idiopathic preterm birth; TB, uncomplicated term births. T) Stacked bar plot showing the iTrophoblast subclusters for each sample in the GSE148241 dataset^[3]^ deconvolution by BayesPrism. EOSPE, early-onset severe preeclampsia.


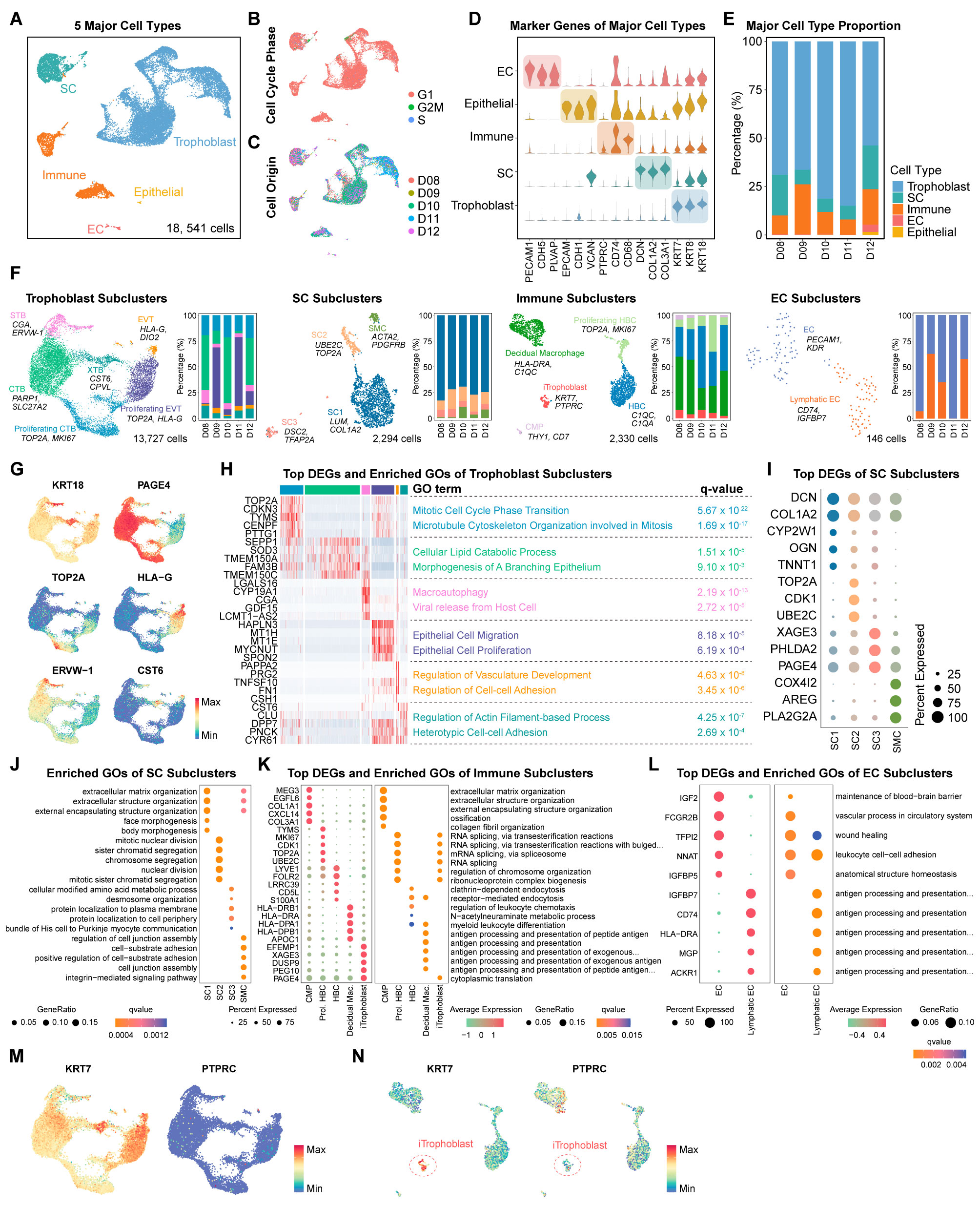


**Figure S6.** Reconstruction of single-cell transcriptome profiles at the human first-trimester maternal-fetal interface.^[4]^ A) UMAP visualization showing five major cell types. B) UMAP visualization showing the cell cycle phase of human placental cells. C) UMAP visualization showing the origin of placental cells in different samples. D) Violin plot showing expression of classical marker genes in major cell types. E) Stacked bar plot showing the proportion of major cell types in five samples. F) UMAP visualization showing subclusters of human placental cell types, with a stacked bar plot showing the proportion corresponding to each subcluster. G) UMAP visualization showing marker gene expression in different placental trophoblast subclusters. H) Heatmap showing the top DEGs of trophoblast subclusters, and the GO biological process terms enriched by corresponding subcluster DEGs. I) Dot plot showing the expression of top DEGs in SC subclusters. J) GO enrichment results of SC subclusters. K) Dot plot showing the top DEGs in immune cell subclusters and the enriched GO terms. L) Dot plot showing the top DEGs in EC subclusters and the enriched GO terms. M) UMAP visualization showing typical marker gene expression of trophoblast (*KRT7*) and immune (*PTPRC*) cells in clustered trophoblast cell subclusters. N) UMAP visualization showing typical marker gene expression of trophoblast (*KRT7*) and immune (*PTPRC*) cells in clustered immune cell subclusters.


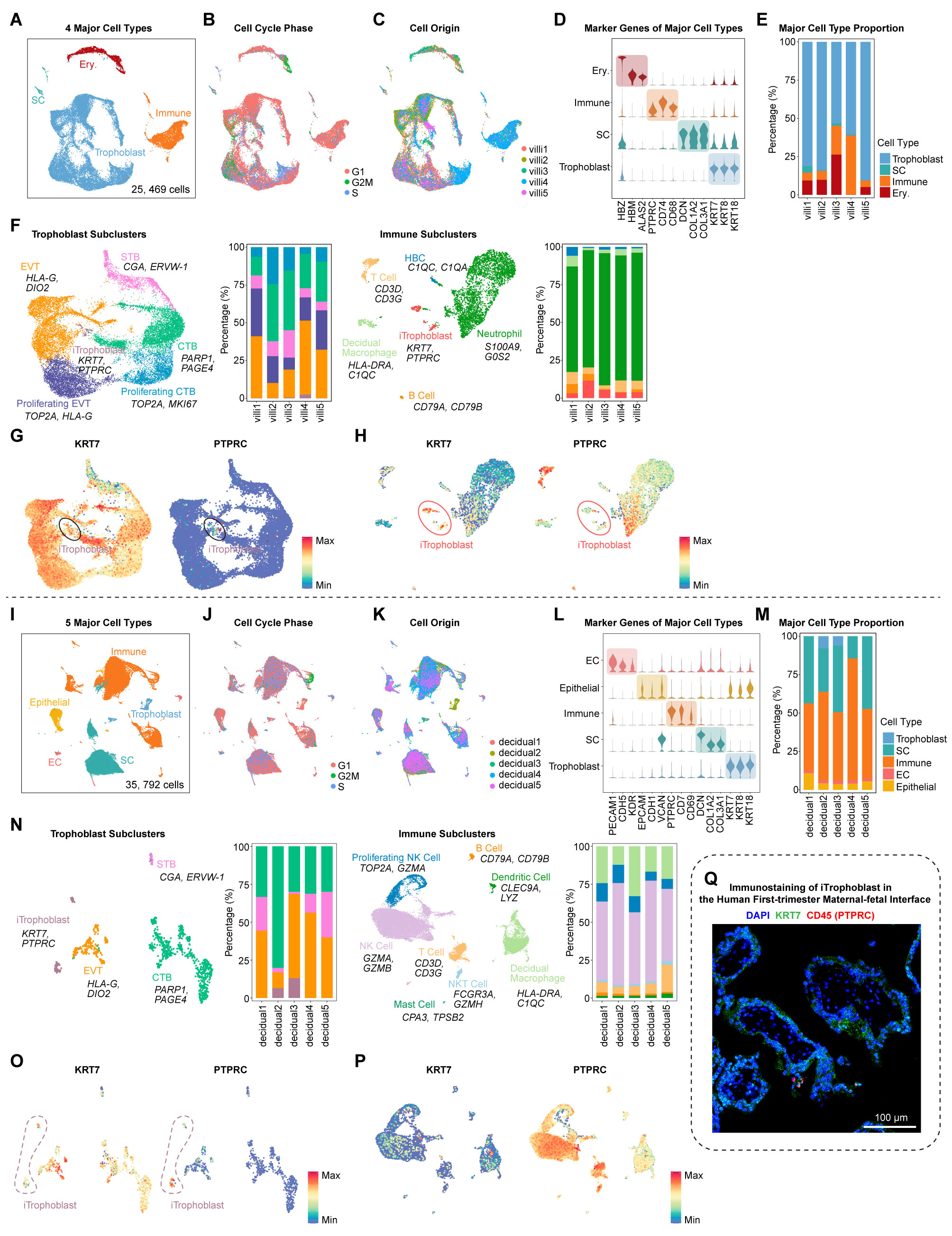


**Figure S7.** Reconstruction of single-cell transcriptome profiles at the human first-trimester maternal-fetal interface.^[5]^ A-H) Placental villi. A) UMAP visualization showing four major cell types. B) UMAP visualization showing the cell cycle phase of human placental villi cells. C) UMAP visualization showing the origin of placental villi cells in different samples. D) Violin plot showing expression of classical marker genes in major cell types. E) Stacked bar plot showing the proportion of major cell types in five samples. F) UMAP visualization showing subclusters of human placental villi cell types, with a stacked bar plot showing the proportion corresponding to each subcluster. G) UMAP visualization showing typical marker gene expression of trophoblast (*KRT7*) and immune (*PTPRC*) cells in clustered trophoblast cell subclusters. H) UMAP visualization showing typical marker gene expression of trophoblast (*KRT7*) and immune (*PTPRC*) cells in clustered immune cell subclusters. I-P) Placental decidua. I) UMAP visualization showing five major cell types. J) UMAP visualization showing the cell cycle phase of human placental decidual cells. K) UMAP visualization showing the origin of placental decidual cells in different samples. L) Violin plot showing expression of classical marker genes in major cell types. M) Stacked bar plot showing the proportion of major cell types in five samples. N) UMAP visualization showing subclusters of human placental decidual cell types, with a stacked bar plot showing the proportion corresponding to each subcluster. O) UMAP visualization showing typical marker gene expression of trophoblast (*KRT7*) and immune (*PTPRC*) cells in clustered trophoblast cell subclusters. P) UMAP visualization showing typical marker gene expression of trophoblast (*KRT7*) and immune (*PTPRC*) cells in clustered immune cell subclusters. Q) Immunostainings of KRT7 and CD45 (PTPRC) protein in the human first-trimester maternal-fetal interface.


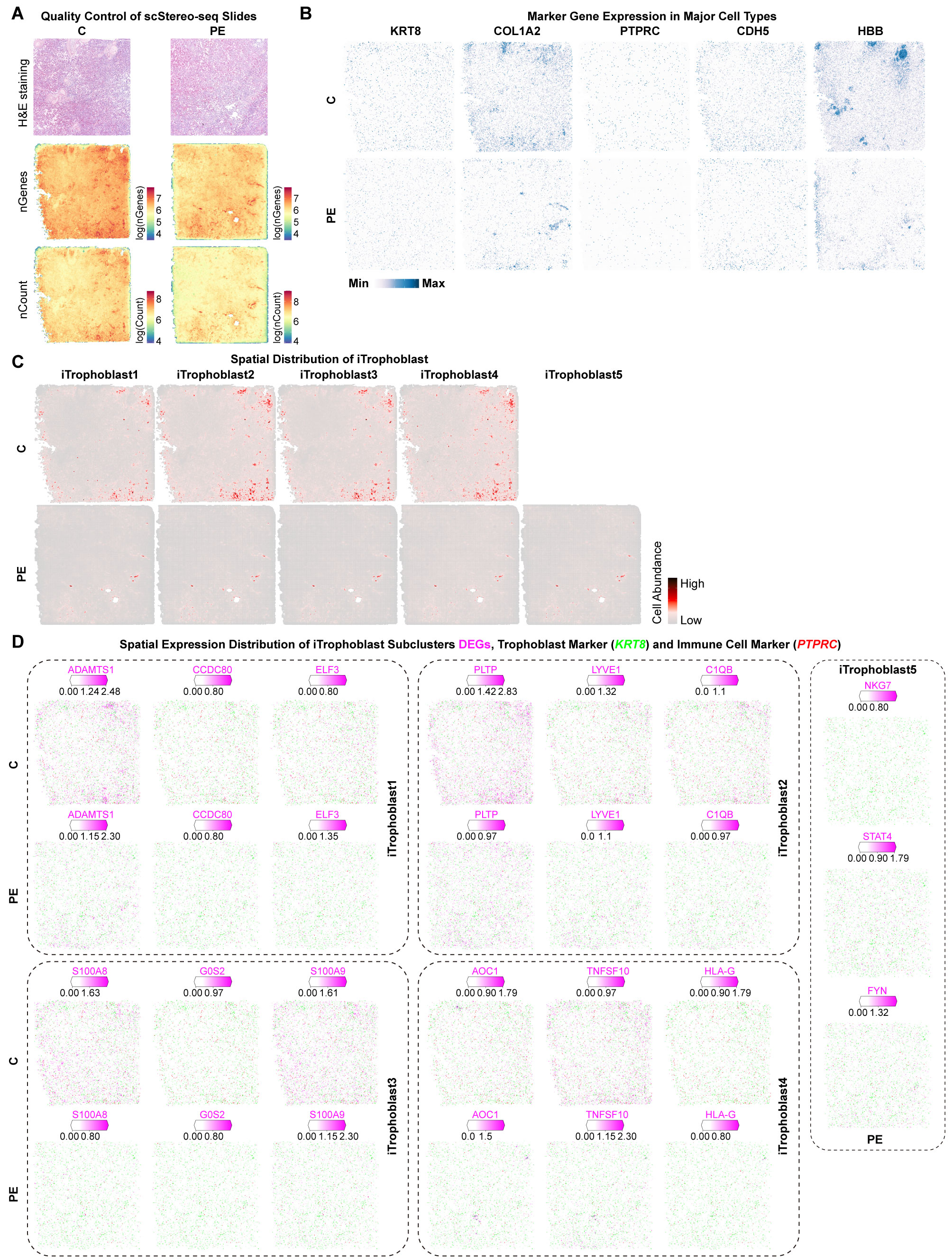


**Figure S8.** Spatial identification and visualization of iTrophoblast in normal and preeclamptic placentas using scStereo-seq. A) Quality check of scStereo-seq data. H&E staining of tissue slides adjacent to those used for scStereo-seq (first row). scStereo-seq spot overlay (bin 100) showing gene numbers (nGenes, second row) and reads (nCount, third row). B) Spatial visualization of gene expression associated with major cell types in placenta. C) Estimated cell abundances (color intensity) of iTrophoblast subclusters across regions. D) scStereo-seq heatmaps showing spatial expression of iTrophoblast subcluster DEGs, *KRT8* and *PTPRC*.


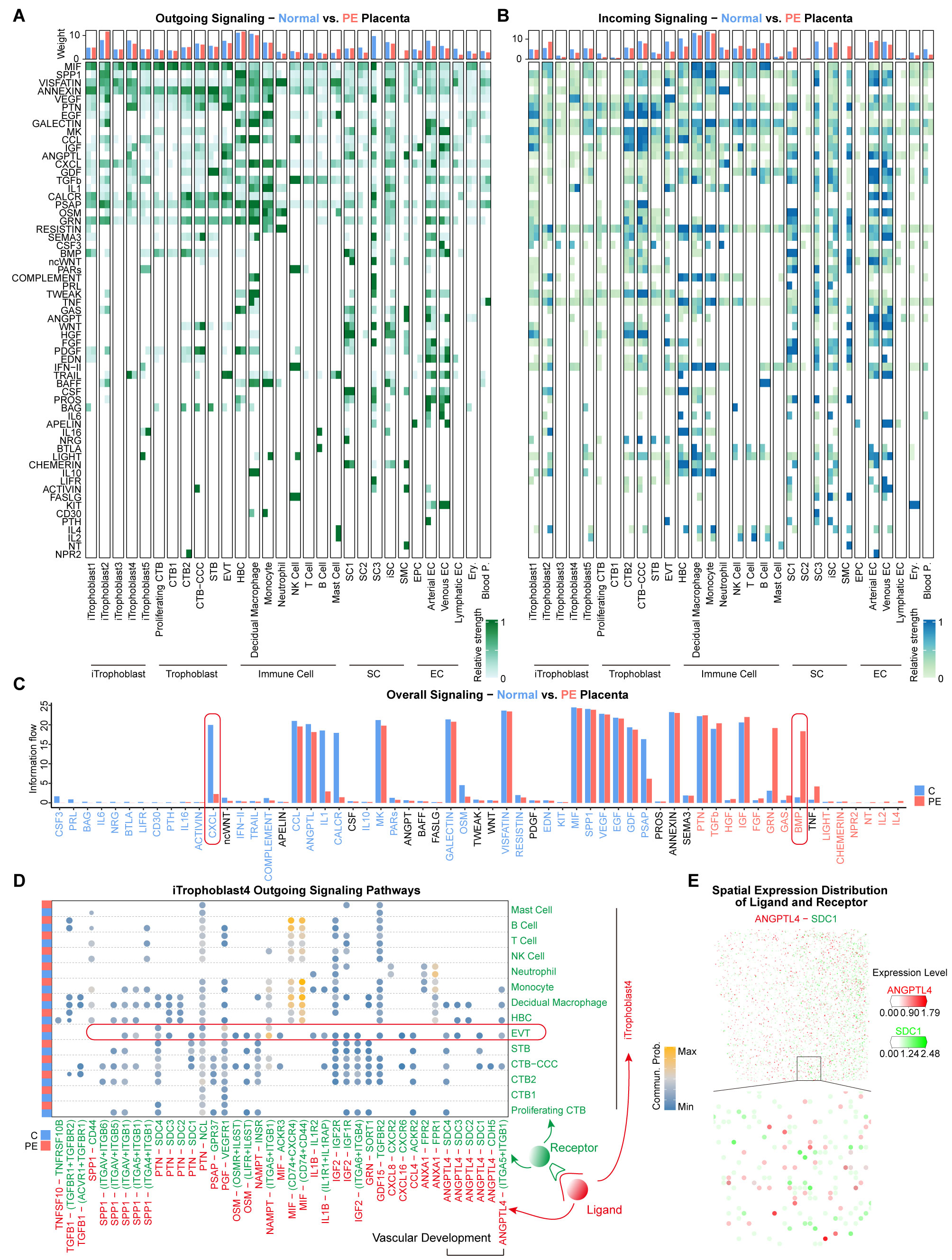


**Figure S9.** Dysfunctional and spatially disorganized iTrophoblast leads to failure of placental EVT invasion and preeclampsia. A and B) Heatmap showing the relative strength of outgoing (A) and incoming (B) signals in each cell subcluster in PE placenta compared to normal placenta. The bar plot above showing the relative strength of overall signals in each cell subcluster. C) Bar plot showing the overall strength of each signaling pathway in PE placenta compared to normal placenta. D) Dot plot showing outgoing signals from the iTrophoblast4 subcluster emitted to trophoblast and immune cell subclusters. E) scStereo-seq heatmaps of normal placenta showing spatial expression of *ANGPTL4* and *SDC1*.

References

[1] X. Jiang, Y. Wang, Z. Xiao, L. Yan, S. Guo, Y. Wang, H. Wu, X. Zhao, X. Lu,H. Wang, *Cell Discovery* **2023**, *9*, 30.

[2] W. E. Ackerman Iv, C. S. Buhimschi, T. L. Brown, G. Zhao, T. L. Summerfield,I. A. Buhimschi, *Hypertension* **2023**, *80*, 1363.

[3] X. Yang, J. Yang, X. Liang, Q. Chen, S. Jiang, H. Liu, Y. Gao, Z. Ren, Y.-W. Shi,S. Li, *Hypertension* **2020**, *75*, 1532.

[4] R. Vento-Tormo, M. Efremova, R. A. Botting, M. Y. Turco, M. Vento-Tormo, K. B. Meyer, J.-E. Park, E. Stephenson, K. Polański,A. Goncalves, *Nature* **2018**, *563*, 347.

[5] P. Wei, M. Dong, Y. Bi, S. Chen, W. Huang, T. Li, B. Liu, X. Fu,Y. Yang, *Frontiers in Immunology* **2022**, *13*, 1053819.
